# The Nuclear Farnesoid X Receptor Reduces p53 Ubiquitination and Inhibits Cervical Cancer Cell Proliferation

**DOI:** 10.3389/fcell.2021.583146

**Published:** 2021-04-06

**Authors:** Xiaohua Huang, Bin Wang, Runji Chen, Shuping Zhong, Fenfei Gao, Yanmei Zhang, Yongdong Niu, Congzhu Li, Ganggang Shi

**Affiliations:** ^1^Department of Pediatrics, Second Affiliated Hospital of Shantou University Medical College, Shantou, China; ^2^Department of Pharmacology, Shantou University Medical College, Shantou, China; ^3^Department of Biochemistry and Molecular Biology, University of Southern California, Los Angeles, Los Angeles, CA, United States; ^4^Department of Gynecology, Cancer Hospital, Shantou University Medical College, Shantou, China

**Keywords:** FXR, cervical cancer, p53, MDM2, ubiquitination

## Abstract

The role of farnesoid X receptor (FXR) in cervical cancer and the underlying molecular mechanism remain largely unknown. Therefore, this study aimed to assess the mechanism of FXR in cervical cancer. Western blot, qRT-PCR, and immunohistochemistry demonstrated that FXR was significantly reduced in squamous cell carcinoma tissues, although there were no associations of metastasis and TNM stage with FXR. In Lenti-FXR cells obtained by lentiviral transfection, the overexpression of FXR reduced cell viability and colony formation. Compared with the Lenti-Vector groups, the overexpression of FXR induced early and late apoptosis and promoted G1 arrest. With time, early apoptosis decreased, and late apoptosis increased. In tumor xenograft experiments, overexpression of FXR upregulated small heterodimer partner (SHP), murine double minute-2 (MDM2), and p53 in the nucleus. Co-immunoprecipitation (Co-IP) showed that SHP directly interacted with MDM2, which is important to protect p53 from ubiquitination. Nutlin3a increased MDM2 and p53 amounts in the Lenti-Vector groups, without effects in the Lenti-FXR groups. Silencing SHP reduced MDM2 and p53 levels in the Lenti-FXR groups, and Nutlin3a counteracted these effects. Taken together, these findings suggest that FXR inhibits cervical cancer via upregulation of SHP, MDM2, and p53.

## Introduction

Cervical cancer is the third most common and deadliest gynecologic malignancy in developed countries (Siegel et al., 2020). In underdeveloped countries, cervical cancer is the second most common malignancy and the third leading cause of cancer-related deaths in women (Vineis and Wild, 2014). Cervical cancer comprise 80% squamous cell carcinoma and 9% cervical adenocarcinoma cases (Lee et al., 2015). A recent meta-analysis revealed that bevacizumab combined with paclitaxel-topotecan or paclitaxel-cisplatin likely prolongs overall survival (OS) compared with regimens not including bevacizumab, with high efficacy in patients with advanced, persistent, and recurrent cervical cancer (Rosen et al., 2017). Nevertheless, resistance to chemotherapy is common, and new treatments are needed to improve cervical cancer prognosis.

Nuclear receptors are ligand-activated transcription factors comprising 48 members, including farnesoid X receptor (FXR), which is activated by bile acids (BAs) such as chenodeoxycholic acid (CDCA), lithocholic acid (LCA), deoxycholic acid (DCA), and cholic acid (CA) (Ding et al., 2015). CDCA is the most effective physiological ligand of FXR (Jia et al., 2014), which is highly expressed in the liver (He et al., 2015), kidney (Zhu et al., 2018), and adrenal gland (Huang et al., 2014), but also in the cervix (Anaya-Hernandez et al., 2014). The order of potency of the BAs to bind FXR is CDCA > LCA = DCA > CA (Ding et al., 2015). In addition to its role in liver regeneration and inflammation, FXR also regulates tumorigenesis and proliferation of hepatocellular carcinoma (HCC) (Huang et al., 2015), pancreatic cancer (Giaginis et al., 2015), and gastric cancer (Duan and Fang, 2014). Interestingly, spontaneous HCC in FXR-null mice can be prevented by intestinal FXR reactivation (Degirolamo et al., 2015). In addition, FXR agonists such as CDCA and GW4064 induce the apoptosis of breast cancer cells and activate the FXR-small heterodimer partner (SHP)-liver receptor homolog-1 (LRH-1) network (Swales et al., 2006).

In previous studies, FXR has been associated with sex hormone-related tumors. GW4064, a specific FXR agonist, significantly reduces the growth of Leydig tumor by inhibiting cell proliferation, inducing apoptosis, and upregulating p53. The anti-proliferative effect of FXR on Leydig cells is partly due to the inhibition of estrogen-dependent cell growth (Catalano et al., 2010, 2013). Furthermore, FXR is a negative regulator of the androgen-estrogen-converting aromatase enzyme in breast cancer cells. The high expression of FXR is significantly correlated with the decrease of tumor volume and proliferation rate of breast cancer, which is a powerful independent predictor of overall and disease-free survival in invasive breast cancer (Giaginis et al., 2017). The activation of FXR inhibits prostate cancer cells through the SREBP1 pathway, thus significantly inhibiting cell proliferation (Liu et al., 2016). Nevertheless, the role of FXR in cervical cancer remains largely unclear.

FXR regulates its target genes through many mechanisms, including SHP activation (Goodwin et al., 2000). Meanwhile, SHP plays an important role in metabolic diseases (Ding et al., 2015), including hyperlipidemia, diabetes, and liver fibrosis (Weiskirchen and Tacke, 2015), and SHP^–/–^ mice have a higher incidence of spontaneous HCC (Zhang et al., 2008). Increasing evidence suggests that SHP inhibits HCC by suppressing proliferation (Wen et al., 2018) and promoting apoptosis (Tai et al., 2012). Notably, the FXR/SHP pathway plays an important role in maintaining BA balance (Kim et al., 2017). This effect mainly depends on the regulatory role of SHP on multiple metabolic genes (Zhang et al., 2011; Seok et al., 2013). In cancer, SHP stabilizes MDM2 protein by preventing its ubiquitination (Yang and Wang, 2012).

One of the LRH-1 co-activators associated with SHP activity is FXR. LRH-1 exists in the endoderm and gonad and is essential for the development beyond gastrulation. LRH-1 can prevent ovulation, cumulus expansion, and luteinization. LRH-1 regulates cell proliferation, migration, invasion, and chemoresistance in breast cancer cells (Meinsohn et al., 2019).

MDM2, also referred to as E3 ubiquitin-protein ligase, is a zinc finger protein that can be inactivated by interaction with the tumor suppressor p53 (Wade et al., 2013; Inoue et al., 2016). Indeed, MDM2 is an important negative regulator of p53 and promotes its degradation by direct combination via an N-terminal domain to form the MDM2-p53 complex (Khoo et al., 2014). MDM2 is overexpressed in multiple human tumors and is therefore considered an oncogene (Zhao et al., 2014). Nutlin3a, a highly specific MDM2 antagonist, inhibits MDM2 binding to p53 (Azer, 2018).

Despite this immense wealth of knowledge, the role of FXR in cervical cancer and the underlying molecular mechanism remain largely unknown. Therefore, the present study aimed to assess the role and mechanism of FXR in cervical cancer. We demonstrated that FXR suppresses cervical cancer by upregulating SHP, MDM2, and p53 via direct binding to SHP through the LRH-1 binding site, SHP-MDM2 complex formation, and p53 ubiquitination blockage.

## Materials and Methods

### Cell Culture

HeLa and CaSki cells were purchased from the American Type Culture Collection (ATCC, United States). SiHa cells were obtained from the Cell Bank of Typical Culture Preservation Committee of the Chinese Academy of Sciences (China). All cells were cultured in Dulbecco’s modified Eagle’s medium (DMEM; Life Technologies, United States) containing 10% fetal bovine serum (Biowest, United States) and 100 U/ml penicillin-streptomycin (Beyotime, China) at 37°C in a humid incubator with 5% CO_2_. The medium was replaced every 2 days. Cells were authenticated by morphological assessment and short tandem repeat (STR) analysis.

### Cervical Cancer Tissue Specimens

Fifty-six cervical cancer tissue specimens were obtained from Shantou University Medical College Cancer Hospital (Supplementary Table 1). A tissue microarray containing 165 tissue samples was purchased from Alenabio (China; Supplementary Table 2). The clinical specimens were graded according to the 2009 FIGO clinical staging system for cervical cancer. The studies involving human participants were reviewed and approved by the ethics committee of the Second Affiliated Hospital of Shantou University Medical College (2015-008). The patients provided written informed consent to participate in this study. The study was carried out in accordance with the World Medical Association Declaration of Helsinki.

### RNA Extraction and Real-Time Quantitative PCR

Details are shown in the Supplementary Materials. The primer sequences are shown in Supplementary Table 3.

### Western Blot

Details are shown in the Supplementary Materials. They were detected using mouse anti-β-actin, mouse anti-MDM2, mouse anti-p53, rabbit anti-FXR, and mouse anti-SHP primary antibodies (Supplementary Table 4).

### Immunohistochemistry

Details are shown in the Supplementary Materials. The tissue sections were incubated with mouse anti-MDM2, mouse anti-p53, and rabbit anti-FXR primary antibodies (Supplementary Table 4).

### Lentivirus-Mediated Transfection

A lentivirus encoding FXR (Genechem, China) was transfected into CaSki, SiHa, and HeLa cells. The transfection medium was changed after 12 h, and puromycin-resistant cells stably overexpressing FXR were selected and confirmed by western blot.

### Cell Proliferation

Cells were inoculated at a density of 3 × 10^3^ cells/well in 96-well plates. CA (100 μg/mL) (Chong et al., 2010), LCA (40 μmol/L) (Hoeke et al., 2014), CDCA (50 μmol/L) (Manfredi, 2010; Marine and Lozano, 2010), and GW4064 (2 μmol/L) (Marine and Lozano, 2010) were added to the medium, respectively, and cell viability was measured by the MTT (Sigma, United States) assay at 24, 48, and 72 h. In addition, the Lenti-FXR and Lenti-Vector groups of CaSki, SiHa, and HeLa cells were assessed for viability as described above. Details are shown in the Supplementary Materials.

### Colony Formation Assay

Cells were inoculated at 500 cells/ml/well, administered CDCA (50 μmol/l) and incubated for 2 weeks. The colony formation rate was assessed as (colony number/cell inoculation number) × 100%. Details are shown in the Supplementary Materials.

### Flow Cytometry

Cells (1 × 10^6^) were harvested, incubated with propidium iodide (PI, Sigma, United States) and RNase A, and analyzed on a BD Accuri^TM^ C. Details are shown in the Supplementary Materials.

### Apoptosis Assessment

Cells were collected, stained with Annexin-V-FITC (0.25 μg/ml, Dojindo, Japan) and PI, analyzed by flow cytometry. Details are shown in the Supplementary Materials.

### Tumor Xenograft Experiments

Female BALB/c-nude mice (6–8 weeks old) were obtained from the Beijing Vital River Laboratory Animal Technology and housed in specific pathogen-free (SPF) rooms maintained at a constant temperature (22–25°C) and humidity (40–50%). The mice were randomly divided into groups of eight animals. CaSki, SiHa, and HeLa cells (1 × 10^6^) were injected subcutaneously on each mouse’s back. Lenti-FXR and Lenti-Vector cells were injected on the right and left sides, respectively. Tumor size and weight were monitored every 3 days. Tumor volume was calculated by the following formula: V = (length × width^2^)/2. The tumors were weighed and used for RNA or protein extraction after sacrifice.

In another experiment, 2 × 10^6^ Siha, CaSki, and Hela cells, respectively, were injected subcutaneously into nude mice (Dai et al., 2011). Eighteen days later, tumor volumes reached about 40 mm^3^. The mice were randomized into two groups. In the control and CDCA groups, the animals were intraperitoneally injected with 100 μl DMSO and CDCA (130 mg/kg), respectively. After treatment, all mice were kept under a laminar flow cabinet for 6 days. All animals received humane treatment according to institutional policies, and the animal study was reviewed and approved by the animal ethics committee of Shantou University Medical College (SUMC 2015-006).

### Reporter Assay

The activities of the Firefly and Renilla luciferase reporters were measured with the Dual Luciferase Assay kit (Promega, United States). Details are shown in the Supplementary Materials.

### DNA Pull-Down Assay

DNA probes and nucleus extracts were resuspended with magnetic beads and incubated for 1 h at 4°C. FXR was precipitated with a 68-bp biotin-labeled DNA probe containing “wild type” (presumed LRH-1 binding site expressed in italics) or “mutant” (mutation in presumed LRH-1 binding site) (Supplementary Figure 1B). Biotin-labeled DNA probes, including IR-1 (TGT CAC TGA ACT GTG CTT GGG CTG CCC TTA GGG ACA TTG ATC CTT AGG CAA AT) or LacI (GTA GTG GCG AAA TTG TGA GCG CTC ACA ATT CGT TTG GCC G) promoter fragments, were used as positive and negative controls, respectively. In competitive experiments, the nuclear extracts were pre-incubated for 1 h at 4°C with three times of unlabeled “wild type” or “mutant type”. DNA binding was analyzed by western blot with anti-FXR antibodies.

### Vector Transfection

Details are shown in the Supplementary Materials.

### Co-immunoprecipitation

HEK293T cells were transfected and anti-FLAG M2 magnetic beads (Sigma, M8823, United States; 50 μl/sample) were used for immunoprecipitation including rabbit anti-MDM2, rabbit anti-p53, rabbit anti-FXR, and rabbit anti-ubiquitin (Supplementary Table 7).

In another assay, the supernatants of Lenti-FXR CaSki cells were incubated with 2 μg IgG or mouse anti-SHP (Santa Cruz, CA, United States). The mixture was incubated with protein G-Agarose beads (Roche, United States) and the samples were assessed by western blot.

Details are shown in the Supplementary Materials.

### Immunofluorescence

Details are shown in the Supplementary Materials. The sections were incubated with mouse anti-MDM2, mouse anti-p53, and mouse anti-SHP primary antibodies, respectively (Supplementary Table 4).

### Protein Ubiquitination

SiHa cells were transfected with Flag-p53 and Myc-MDM2, respectively, and immunoprecipitated. At 48 h post-transfection, the cells were treated with 10 μmol/l MG132 for 2 h. After centrifugation, the supernatants were collected and immunoprecipitated with 20 μl ubiquitin beads (ab7780, Abcam, United States). Western blot with rabbit anti-p53 (Supplementary Table 7) was performed to detect ubiquitinated p53.

SiHa cells were transiently transfected with pcDNA3.1-SHP (Yanjin Biology, China) primers (Supplementary Table 8) using the Lipofectamine 2000. After 48 h, protein ubiquitination was assessed.

In addition, SiHa cells were cultured in a culture medium containing CDCA (50 μmol/l) for 48 h, followed by protein ubiquitination assessment as described above.

### Statistical Analysis

All experiments were repeated three times. One-way analysis of variance (ANOVA) was performed with the SPSS 22.0 statistical software (SPSS). *P* < 0.05 indicated a statistically significant difference.

## Results

### FXR Expression in Human Cervical Cancer

**FIGURE 1:**
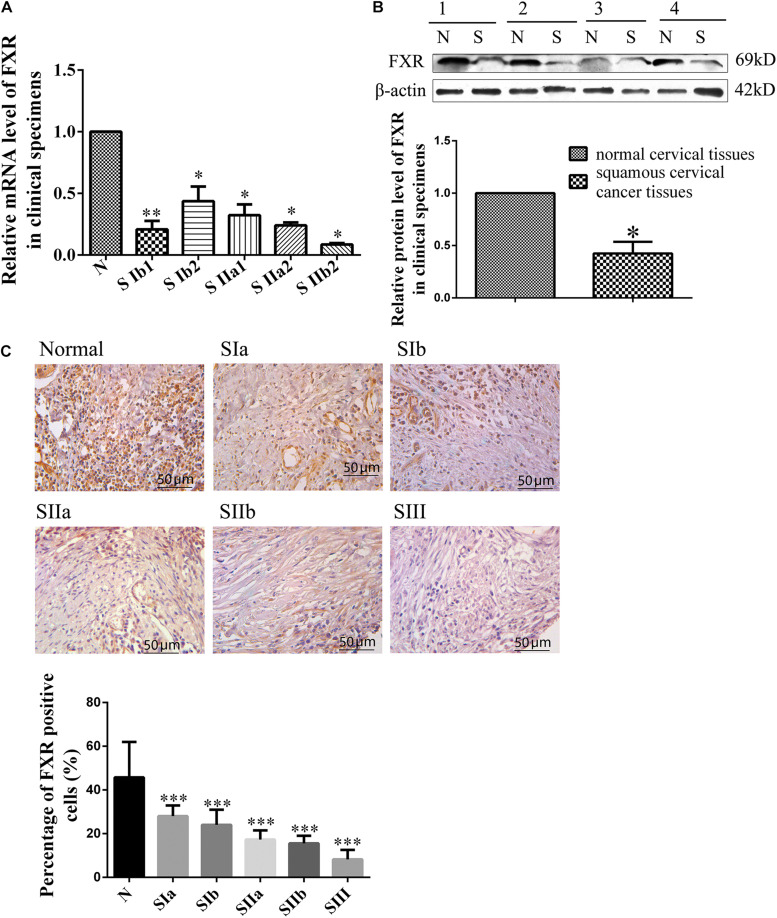
FXR expression in human cervical cancer and non-cancerous tissues. Relative mRNA **(A)** and protein **(B)** levels of FXR in normal and squamous cervical cancer tissues. SIb1, SIb2, SIIa1, SIIa2, and SIIb2 are stages of squamous carcinoma; N represents normal cervical tissue. β-actin was used for normalization. Immunohistochemical staining **(C)** of a tissue microarray (normal and squamous cervical cancer tissues) for FXR. SIa, SIb, SIIa, SIIb, and SIII are stages of squamous carcinoma; N represents normal cervical tissue. Scale bar, 50 μm. ^∗^*P* < 0.05; ^∗∗^*P* < 0.01; ^∗∗∗^*P* < 0.001 vs. normal cervical tissues.

FXR expression was examined in human normal and cancerous cervical tissue samples to determine whether FXR is involved in cervical carcinogenesis (Supplementary Table 1). First, qRT-PCR was performed to detect FXR mRNA levels in 37 cervical cancer and 19 normal cervical tissue specimens (Figure 1A). The results showed that FXR mRNA levels were three times lower in squamous cervical cancer than normal tissue (*p* < 0.05). To confirm FXR expression in cervical carcinogenesis, western blot and immunohistochemistry were carried out. Western blot was used to assess 20 cervical cancer and five normal tissue samples. As shown in Figure 1B, FXR protein amounts in normal cervical tissues were approximately two-fold greater than those of cervical cancer (*p* < 0.05). Immunohistochemistry of a tissue microarray containing 165 pathological specimens (Supplementary Table 2) showed that the FXR positive cell rates in the normal cervix were approximately three-fold higher than those of cervical cancer (Figure 1C, *p* < 0.01). Taken together, these results suggest an inverse correlation between FXR expression and cancerous status. FXR was significantly reduced in squamous cell carcinoma tissues, although there was no connection between metastasis or TNM stage with FXR.

### FXR Inhibits Proliferation in Cervical Cancer Cell Lines *in vitro* by Inducing G1 Arrest and Apoptosis

**FIGURE 2:**
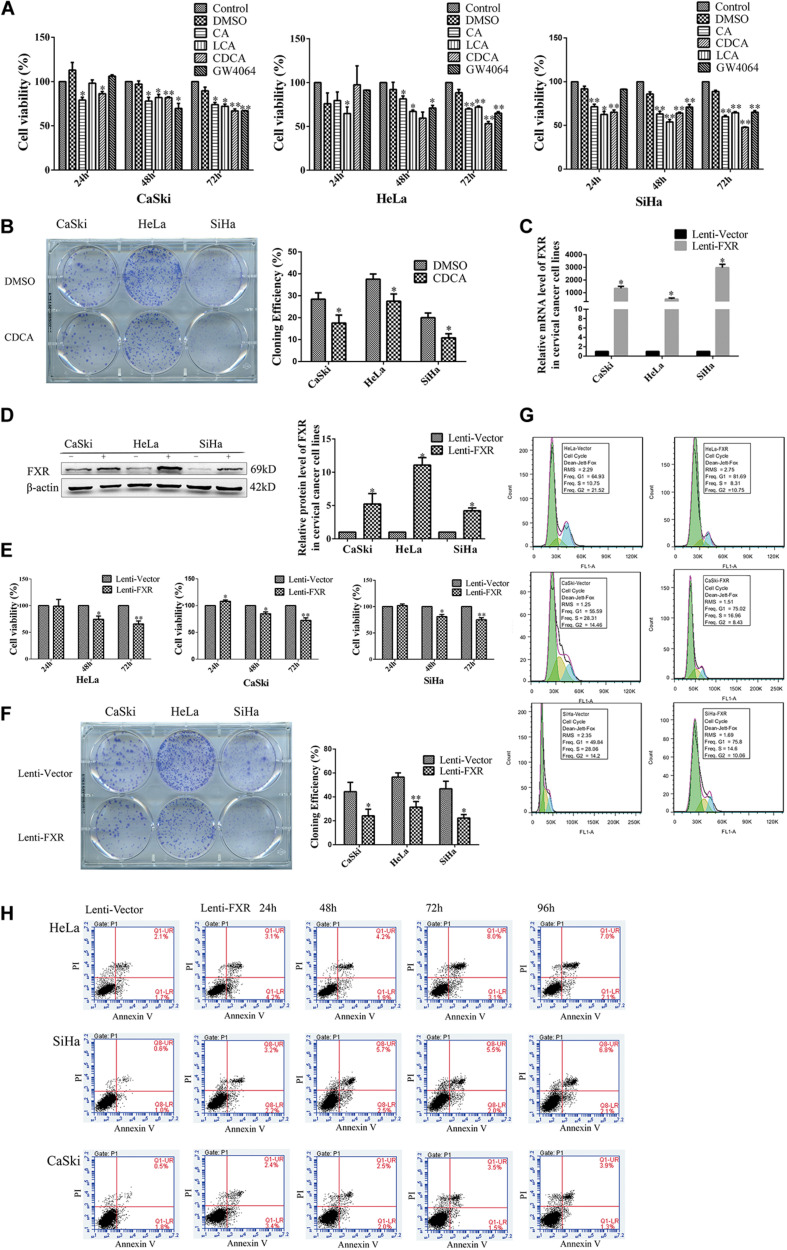
FXR inhibits cervical cancer cell proliferation, inducing G1 arrest and apoptosis. MTT assay of CaSki, HeLa, and SiHa cells following treatment with CA (100 μg/ml), LCA (40 μmol/l), CDCA (50 μmol/l), and GW4064 (2 μmol/l) **(A)**. Colony formation of CaSki, HeLa, and SiHa cells treated with CDCA (50 μmol/l) **(B)**. ^∗^*P* < 0.05; ^∗∗^*P* < 0.01 vs. DMSO group. CaSki, HeLa, and SiHa cells stably overexpressing FXR were confirmed by qRT-PCR **(C)** and western blot **(D)**. Lenti-FXR groups showed reduced proliferation in MTT **(E)** and colony formation **(F)** assays, with G1 arrest [fluorescence; **(G)**] and apoptosis [flow cytometry; **(H)**]. Green, G1; yellow, S; blue, G2. ^∗^*P* < 0.05; ^∗∗^*P* < 0.01 vs. Lenti-Vector group.

In order to confirm a relationship between FXR and cervical cancer, MTT and colony formation assays were performed to assess cervical cancer cell lines following the addition of FXR agonists. As shown in Figure 2A, CA, LCA, CDCA, and GW4064 all reduced cancer cell viability compared with the DMSO control (*p* < 0.05). In addition, colony formation in the CDCA group was lower than that of the DMSO group (Figure 2B, *p* < 0.05).

In order to investigate the relationship between FXR and cervical carcinogenesis, lentiviral transduction was used to engineer FXR-overexpressing cell lines (denoted CaSki-FXR, SiHa-FXR, and HeLa-FXR, respectively). FXR-overexpressing groups (Lenti-FXR) were compared with the vector control (Lenti-Vector) groups by qPCR (Figure 2C, *p* < 0.05) and western blot (Figure 2D, *p* < 0.05). The results showed that FXR mRNA levels in Lenti-FXR groups were >1,000-fold greater than those of Lenti-Vector groups, while FXR protein levels increased by approximately five-fold. As expected, the overexpression of FXR reduced cell viability by 30% (Figure 2E, *p* < 0.05) and colony formation by 50% (Figure 2F, *p* < 0.05) in the MTT and colony formation assays, respectively.

Next, the effects of FXR on cell cycle distribution and apoptosis in CaSki, HeLa, and SiHa cells were measured by flow cytometry. Compared with the values of Lenti-Vector groups, the proportions of cells in the G1 phase in Lenti-FXR groups were significantly higher while the proportions of S/G2 phase cells were decreased (Figure 2G). These results suggested that FXR overexpression inhibited cell proliferation by causing G1 arrest. Compared with the Lenti-Vector groups, the overexpression of FXR induced early and late apoptosis. With time, early apoptosis decreased, and late apoptosis increased (Figure 2H).

### FXR Inhibits Tumor Formation by Cervical Cancer Cells in Nude Mice

**FIGURE 3:**
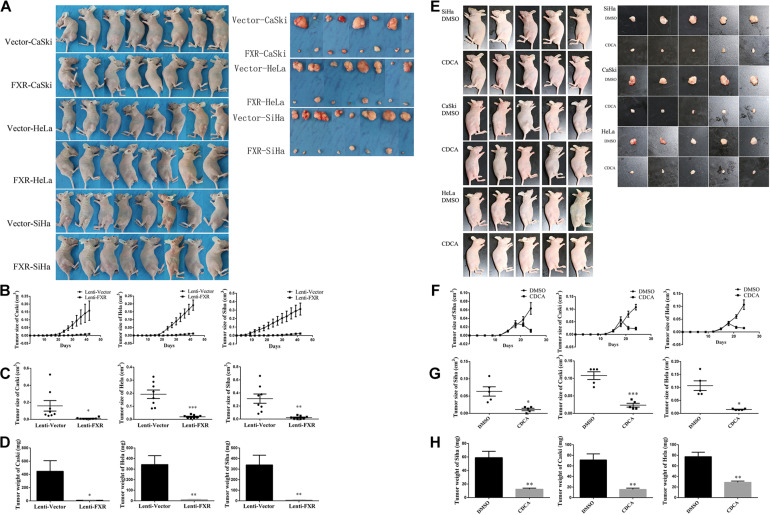
FXR inhibits xenograft tumor formation in cervical cancer cells. **(A)** Female nude mice were injected Vector-CaSki/HeLa/SiHa and FXR-CaSki/HeLa/SiHa cells (*n* = 8). Tumor growth was significantly inhibited by FXR overexpression, as measured by tumor growth **(B)**, volume **(C)**, and weight **(D)** at 42 days after injection. ^∗^*P* < 0.05; ^∗∗^*P* < 0.01; ^∗∗∗^*P* < 0.001 vs. Lenti-Vector group. **(E)** Female nude mice were injected with SiHa, CaSki, or HeLa cells (*n* = 5). Tumor growth was significantly inhibited by FXR activation by CDCA, as measured by tumor growth **(F)**, size **(G)**, and weight **(H)**. ^∗^*P* < 0.05; ^∗∗^*P* < 0.01; ^∗∗∗^*P* < 0.001 vs. DMSO group.

To assess the effect of FXR overexpression on tumor formation *in vivo*, Lenti-FXR and Lenti-Vector cells were injected subcutaneously into nude mice (*n* = 8). As shown in Figure 3A, palpable tumors were observed at 12 days in the Lenti-Vector groups and 18 days in the Lenti-FXR groups. The tumors formed by Lenti-FXR cells grew much slower than those from Lenti-Vector cells (Figure 3B, *p* < 0.05). In agreement, tumor weight and size were reduced in the Lenti-FXR groups compared with the Lenti-Vector groups (Figures 3C,D; *p* < 0.05). These results indicated that FXR upregulation attenuated tumor formation and progression in cervical cancer *in vivo*.

In order to assess whether FXR activation would cause a reduction of tumor growth, treatment of tumor-inoculated nude mice with CDCA was performed (*n* = 5). Interestingly, the administration of CDCA resulted in significantly decreased tumor volumes and weights compared with the control groups (Figures 3E–H).

### FXR Binds to the LRH-1 Binding Site of the SHP Promoter

**FIGURE 4:**
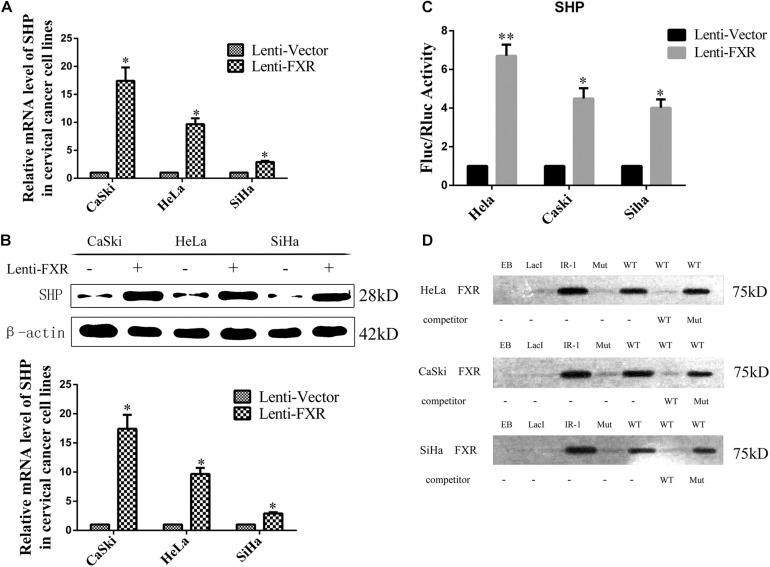
FXR regulates SHP expression through direct binding to the LRH-1 promoter element. Overexpression of FXR increased SHP mRNA **(A)** and protein **(B)** levels. The dual-luciferase activity was measured in the Lenti-Vector or Lenti-FXR transfected cells, the following co-transfection with a SHP promoter reporter vector and a plasmid expressing Renilla luciferase. Fold change was expressed with Renilla luciferase as an internal control **(C)**. A SHP-122/-69 DNA probe binding FXR in DNA pull-down assay **(D)**. ^∗^*P* < 0.05, ^∗∗^*P* < 0.01 vs. Lenti-Vector group.

**FIGURE 5:**
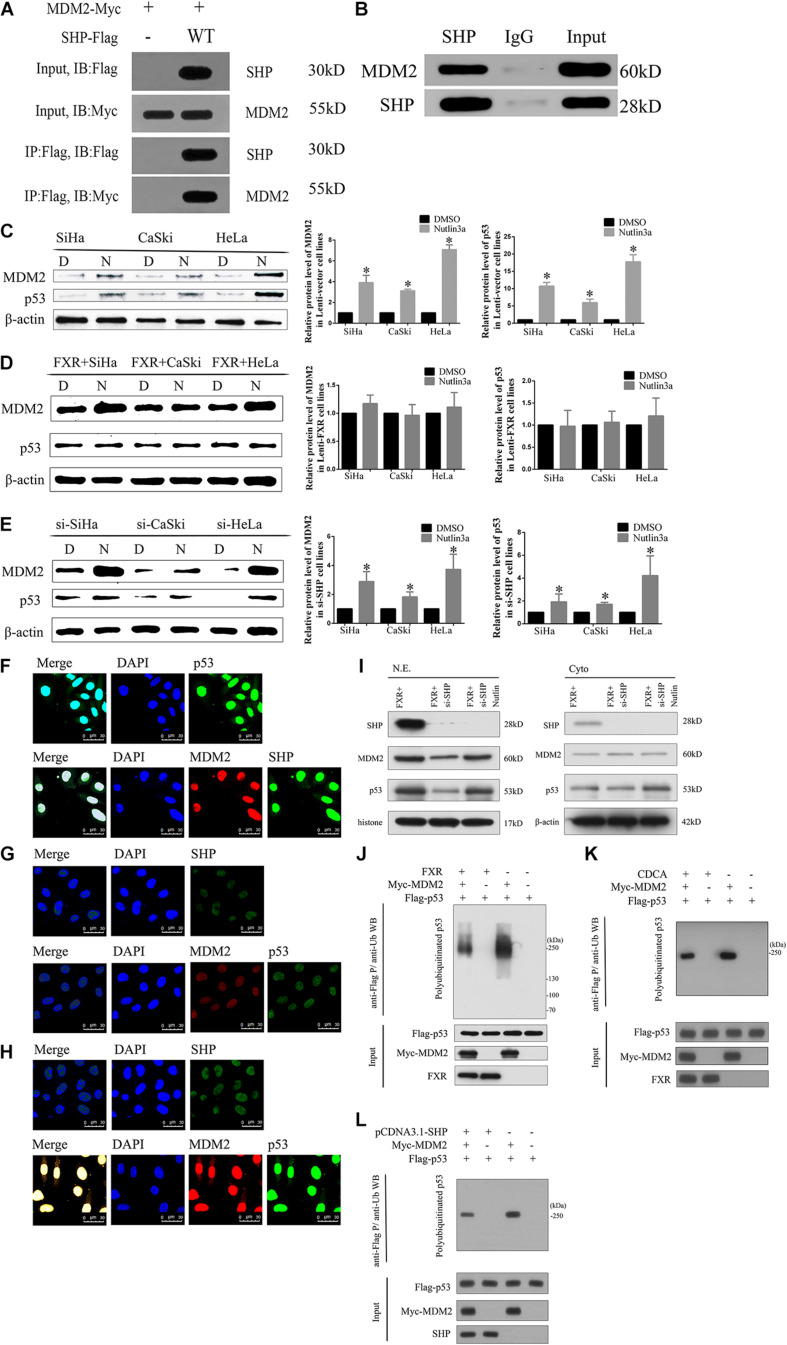
FXR regulates p53 ubiquitination by stabilizing MDM2 aggregation in the nucleus through SHP. **(A)** Immunoprecipitation and western blot were performed to determine the association of SHP-WT with MDM2. An anti-Myc antibody was used to detect MDM2, and the anti-Flag antibody was used to IP SHP. **(B)** The MDM2 protein was immunoprecipitated with polyclonal anti-SHP antibodies in Lenti-FXR cells (IP: SHP), and immunoprecipitates were resolved by SDS-PAGE. Immunoblotting was performed with anti-MDM2 antibodies. Protein levels of MDM2 and p53 with or without application of Nutlin-3a were detected by western blot in Lenti-Vector cells **(C)**, Lenti-FXR cells **(D)**, and Lenti-FXR cells transfected with si-SHP **(E)**. D, DMSO; N, Nutlin3a. Immunofluorescent staining of SHP, MDM2, and p53 in SiHa-FXR cells **(F)** and counterparts co-transfected with si-SHP **(G)** or si-SHP and Nutlin-3a **(H)**. Nuclei (blue) are stained with 4′-6-diamidino-2-phenylindole (DAPI). **(I)** Nuclear and cytoplasmic extracts were obtained for immunoblot analysis of MDM2, p53, and SHP expression. **(J)** SiHa was co-transfected with Flag-p53 and Myc-MDM2; 48 h later, cells were treated with 10 μmol/l MG132 for 2 h and harvested for immunoblot assay with anti-p53 antibody. The SiHa cells were transiently transfected with pCDNA3.1 **(K)** or cultured in the medium containing CDCA (50 μmol/l) **(L)**. After 48 h, the protein ubiquitination was performed. ^∗^*P* < 0.05 vs. DMSO group.

SHP is a downstream target gene of FXR (Zhang et al., 2011). In recent mouse genome-wide ChIP experiments, significant enrichment of LRH-1 binding sites was detected in DNA sequences precipitated with anti-FXR antibodies, indicating FXR and LRH-1 co-induce SHP transcription in mice (Chong et al., 2010). Based on these observations, functional LRH-1 binding sites have been identified in several genes controlled by FXR, including SHP (Goodwin et al., 2000). In this study, overexpression of FXR increased the mRNA (Figure 4A, *p* < 0.05) and protein (Figure 4B, *p* < 0.05) levels of SHP in the nucleus (Figure 5F). The LRH-1 binding site in the SHP promoter is required for FXR-dependent induction of SHP (Hoeke et al., 2014). We engineered a GV238-SHP reporter plasmid containing the SHP promoter LRH-1 binding site driving luciferase expression (Supplementary Figure 1A). When GV238-SHP and a plasmid expressing Renilla luciferase were co-transfected, the Fluc/Rluc activity ratio was higher in FXR-overexpressing cells (Figure 4C). Next, we analyzed whether FXR binds to the -122/-69 region in the SHP promoter directly using a 68 bp biotinylated DNA (SHP promoter-122/-69 region) fragment containing the LRH-1 sequence (Supplementary Figure 1B) in a DNA pull-down assay. FXR effectively pulled down the LRH-1 DNA fragment from the extracts of FXR-overexpressing cells, similar to the IR-1 positive control (Figure 4D). When the LRH-1 site was mutated at -122/-69, the binding to FXR was abolished. Furthermore, mutant sequences did not compete for FXR binding, unlike wild type -122/-69 SHP promoter fragments. These results implied that FXR-mediated activation of SHP was largely regulated by direct binding of FXR to the LRH-1 binding site in the SHP promoter to enhance SHP expression.

### SHP Binds to MDM2 in Cervical Cancer Cell Lines

**FIGURE 6:**
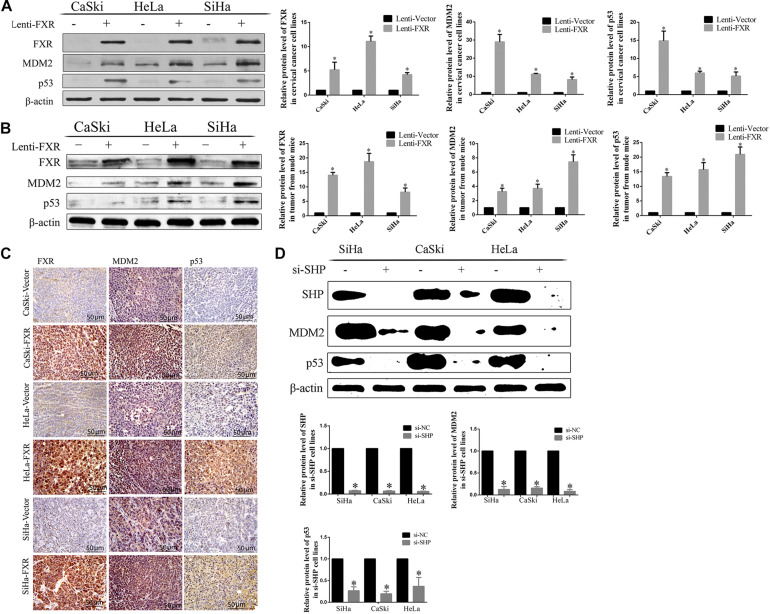
Correlation between FXR and SHP/p53 expression. Overexpression of FXR upregulated MDM2 and p53 in cervical cancer cells **(A)** and xenograft tumors from nude mice, as measured by western blot **(B)** and immunohistochemistry **(C)**. ^∗^*P* < 0.05 vs. Lenti-Vector group. The knockdown of SHP decreased the protein levels of MDM2 and p53 **(D)**. ^∗^*P* < 0.05 vs. si-NC group.

Since MDM2 is degraded by ubiquitination, we hypothesized that SHP might affect the stability of the MDM2 protein by regulating its ubiquitination (Manfredi, 2010; Marine and Lozano, 2010). The expression levels of both SHP (Figure 4B) and MDM2 (Figure 6A) were increased in the nucleus (Figure 5F) of Lenti-FXR cells. Transfection with si-SHP reduced MDM2 protein levels (Figure 6D, *p* < 0.05), suggesting that SHP upregulated MDM2. Co-IP showed that SHP directly interacted with MDM2 (Figures 5A,B) (Yang and Wang, 2012). Therefore, the interaction between SHP and MDM2 is essential for their cross-regulation.

### SHP and MDM2 Interaction Is Important to Protect p53 From Ubiquitination

Nutlin-3a was used as an inhibitor of the ubiquitination ligase E3 to assess the mechanism by which SHP acts on MDM2 to prevent p53 ubiquitination mediated by MDM2. The overexpression of FXR resulted in increased protein levels of MDM2 and p53 *in vitro* (Figure 6A, *p* < 0.05) and *in vivo* (Figures 6B,C, *p* < 0.05) in the nucleus (Figure 5F). MDM2 and p53 were translocated from the nucleus to the cytoplasm (Figures 5F,G), showing decreased amounts in Lenti-FXR cells after SHP knockdown (Figure 6D). The use of si-SHP and Nutlin-3a in Lenti-FXR cells restored MDM2 and p53 protein levels in the nucleus (Figures 5E,H,I). MDM2 and p53 levels were increased in the Nutlin-3a groups compared with the DMSO groups in Lenti-Vector cells (Figure 5C). There were no statistically significant differences in the MDM2 and p53 protein levels (Figure 5D) between the Nutlin-3a and DMSO groups of Lenti-FXR cells. It is well known that MDM2 is involved in the ubiquitination of p53 (Manfredi, 2010; Marine and Lozano, 2010; Yang and Wang, 2012; Wade et al., 2013; Zhao et al., 2014; Inoue et al., 2016; Zanjirband et al., 2016; Azer, 2018). As FXR could decrease the interaction between MDM2 and p53 and increase MDM2 and p53, we tested whether FXR could affect the ubiquitination level of the p53 protein. We confirm that MDM2 induced a dramatic increase in p53 ubiquitination. Co-transfection of FXR and MDM2, overexpression of SHP, or activation of FXR can reduce p53 ubiquitination (Figures 5J–L and Supplementary Figure 2).

## Discussion

This study showed that FXR inhibits cervical cancer by upregulating SHP, MDM2, and p53 via direct interaction with SHP through the LRH-1 binding site, induction of SHP-MDM2 complex formation, and suppression of p53 ubiquitination.

In this study, the activation of FXR by FXR agonists and the stable overexpression of FXR by lentiviral transfection inhibited the proliferation of three cervical cancer cell lines, according to the MTT and clone formation assays. In addition, flow cytometry indicated that the overexpression of FXR induced G1 arrest in all three cell lines. These findings are consistent with the ability of the FXR agonist GW4064 to inhibit the proliferation of HCC cells, also resulting in G1 phase arrest (Guo et al., 2015). This effect might be related to the HPV E6 and E7 proteins, which degrade the tumor suppressor p53 and cause G1 cell cycle arrest (Tian et al., 2018). Specifically, HPV E7 eliminates G1 cell cycle checkpoints and induces genomic instability, which plays an important role in cervical carcinogenesis (Qiao et al., 2018).

It is known that FXR induces apoptosis by activating signaling pathways such as caspase-3 (Martinez et al., 1998), AP-1 (Brady et al., 1996), and Cyclooxygenase 2 pathways (Oshio et al., 2008), thereby inhibiting the proliferation of cancer cells (Vousden and Lane, 2007). As shown above, the overexpression of FXR induced apoptosis in cervical cancer cell lines, which might be related to p53 upregulation. It might be the most critical factor through which p53 inhibits the development of cancer (Vousden and Lane, 2007).

SHP is a recognized FXR target gene (Zhang et al., 2015), and the FXR agonist GW4064 significantly induces SHP expression in rat liver and human hepatocytes (Goodwin et al., 2000). An early study showed that LRH-1 is an effective promoter of SHP, closely related to the liver (Zhang et al., 2011). In addition, the LRH-1 site in the human SHP promoter represents an important site for FXR-mediated expression in DLD-1 cells (Hoeke et al., 2014). As shown above, in cervical cancer cells, FXR regulated SHP by binding LRH-1, corroborating the above findings in other tumors. Overexpression of FXR significantly increased transcriptional activity by binding to the LRH-1 site in the SHP promoter. When other sites beyond the -122/-69 fragment are mutated, the SHP promoter activity can also be reduced, but the effect is less obvious than observed after mutation of the LRH-1 site (Hoeke et al., 2014). DNA pull-down assays also confirmed that overexpressed FXR directly bound to the -122/-69 site of the SHP promoter. Hence, overexpression of FXR increases the transcriptional activity of SHP by binding to the LRH-1 site, in agreement with the above reports.

SHP positively regulates the expression of MDM2 (Yang and Wang, 2012). The above results demonstrated that SHP, MDM2, and p53 protein amounts increased upon FXR overexpression. Conversely, their amounts were decreased by transfection with si-SHP. These findings indicate that SHP mediates FXR-induced upregulation of MDM2 and p53. It is known that MDM2 promotes p53 ubiquitination and degradation by binding to its transcriptional activation domain (Azer, 2018). Combined with the above immunofluorescence data, SHP and MDM2 interaction probably occurred in the nucleus, preventing the binding of MDM2 and p53 and inhibiting ubiquitination.

As shown above, Co-IP experiments indicated a physical interaction between SHP and MDM2 (Figures 5A,B), indicating that SHP regulates MDM2 protein stability. Therefore, the interaction between SHP and MDM2 is essential for their cross-regulation, as reported previously (Yang and Wang, 2012). These results indicate that the binding of SHP to MDM2 is important for the ubiquitination of p53.

MDM2 is an important negative regulator of p53 and promotes their degradation by forming the MDM2-p53 complex (Khoo et al., 2014). Nutlin-3a, an inhibitor of ubiquitination ligase E3, inhibits MDM2-p53 binding and ubiquitination mediated by MDM2 (Zanjirband et al., 2016). In this study, MDM2 and p53 protein amounts increased in Lenti-Vector cells upon the addition of Nutlin-3a. It might be explained by that Nutlin-3a suppresses MDM2 binding to p53, thus inhibiting the translocation and ubiquitination of MDM2 and p53, which subsequently increases MDM2 and p53 amounts. The expression levels of MDM2 and p53 remained unchanged in Lenti-FXR cells following treatment with Nutlin-3a. Overexpression of FXR induces SHP-MDM2 complex formation, thereby reducing MDM2 and p53 binding as well as ubiquitination so that Nutlin-3a’s effects are non-detectable. MDM2 and p53 were downregulated in Lenti-FXR cells following treatment with si-SHP. In addition, SHP-MDM2 complex formation was abrogated upon downregulation of SHP, resulting in the formation of the MDM2-p53 complex, which then translocated to the cytoplasm to promote ubiquitination. As a result, MDM2 and p53 amounts were decreased. Overexpression of FXR also resulted in increased nuclear MDM2 and p53 expression levels following treatment with si-SHP and Nutlin-3a. The SHP-MDM2 complex was abrogated upon downregulation of SHP, but MDM2-p53 complex formation and ubiquitination were also inhibited by Nutlin-3a. Taken together, the above findings indicate that the MDM2-p53 complex is formed in the nucleus and translocates to the cytoplasm to promote ubiquitination when SHP expression is low. In the case of FXR overexpression, SHP is upregulated and plays an important role in increasing MDM2 expression through binding with MDM2 to inhibit MDM2-p53 binding and ubiquitination by forming the SHP-MDM2 complex in the nucleus; this slows down MDM2 degradation and increases the stability of the MDM2 protein. Meanwhile, MDM2 is not free when bound to SHP and not available to bind and ubiquitinate p53, which leads to increased MDM2 and p53 levels. However, this effect is abolished in the case of SHP knockdown, even when FXR is overexpressed.

In conclusion, FXR is significantly reduced in cervical squamous cell carcinoma tissues and inhibits cervical cancer cell proliferation by inducing G1 arrest and apoptosis. In addition, we demonstrated that FXR upregulates SHP, MDM2, and p53 by directly binding to the LRH-1 binding site in the SHP promoter and inducing SHP to form the SHP-MDM2 complex in the nucleus, thereby decreasing free MDM2 amounts and preventing MDM2 and p53 ubiquitination and degradation. Therefore, FXR serves as a tumor suppressor in cervical carcinoma, suggesting that FXR agonists represent potentially effective products to prevent and treat cervical cancer.

## Data Availability Statement

The raw data supporting the conclusions of this article will be made available by the authors, without undue reservation.

## Ethics Statement

The studies involving human participants were reviewed and approved by the Ethics Committee of the Second Affiliated Hospital of Shantou University Medical College (2015-008). The patients/participants provided their written informed consent to participate in this study. The animal study was reviewed and approved by the animal Ethics Committee of Shantou University Medical College (SUMC 2015-006).

## Author Contributions

XH and RC carried out the experiments, analyzed the data, and wrote the first and final draft of the manuscript. BW, SZ, and YN gave many good suggestions about the data processing and provided the experimental materials. FG and YZ conceived the study and analyzed the interpretation. CL provided the specimens. GS designed the study, revised the manuscript, and provided funding. All authors read and approved the final manuscript.

## Conflict of Interest

The authors declare that the research was conducted in the absence of any commercial or financial relationships that could be construed as a potential conflict of interest.

## Supplementary Material

Supplementary Figure 1 **(A)** Schematic of GV238 firefly luciferase vector. **(B)** LRH-1 site in the -122/-69 region of the SHP promoter.

Supplementary Figure 2 The pathway of FXR-mediated p53 upregulation.
