# Supplementary figures and images for: The Nuclear Farnesoid X Receptor Reduces p53 Ubiquitination and Inhibits Cervical Cancer Cell Proliferation
Source: Front Cell Dev Biol. 2021 Apr 6;9:583146. doi: 10.3389/fcell.2021.583146 (PMC8056046; doi:10.3389/fcell.2021.583146)

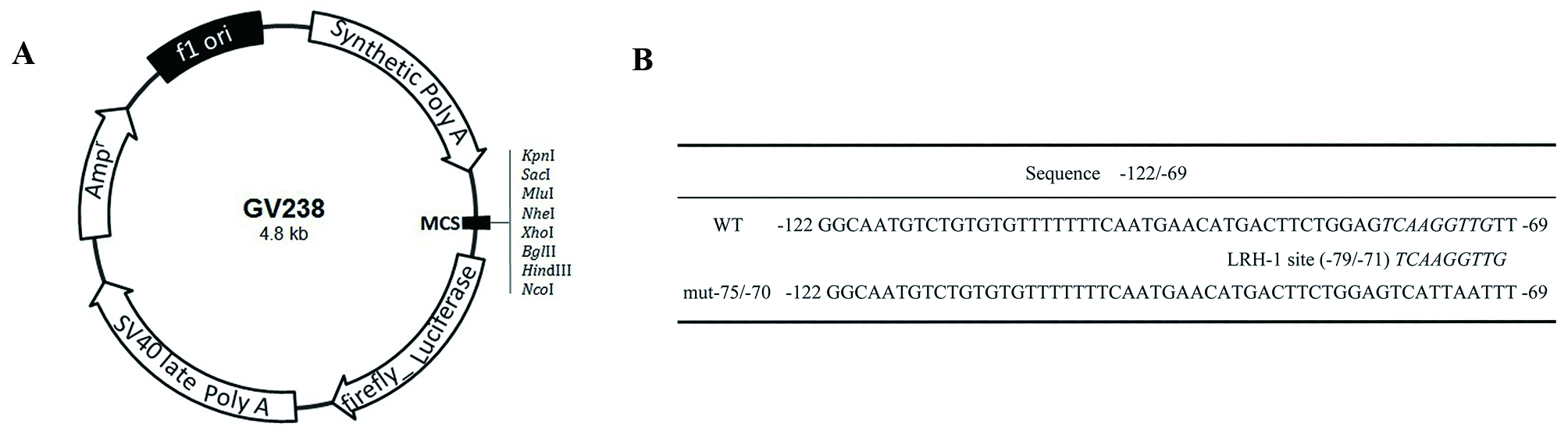

Supplement: Supplementary Figure 1 — (A) Schematic of GV238 firefly luciferase vector. (B) LRH-1 site in the -122/-69 region of the SHP promoter. [file Image_1.TIF]

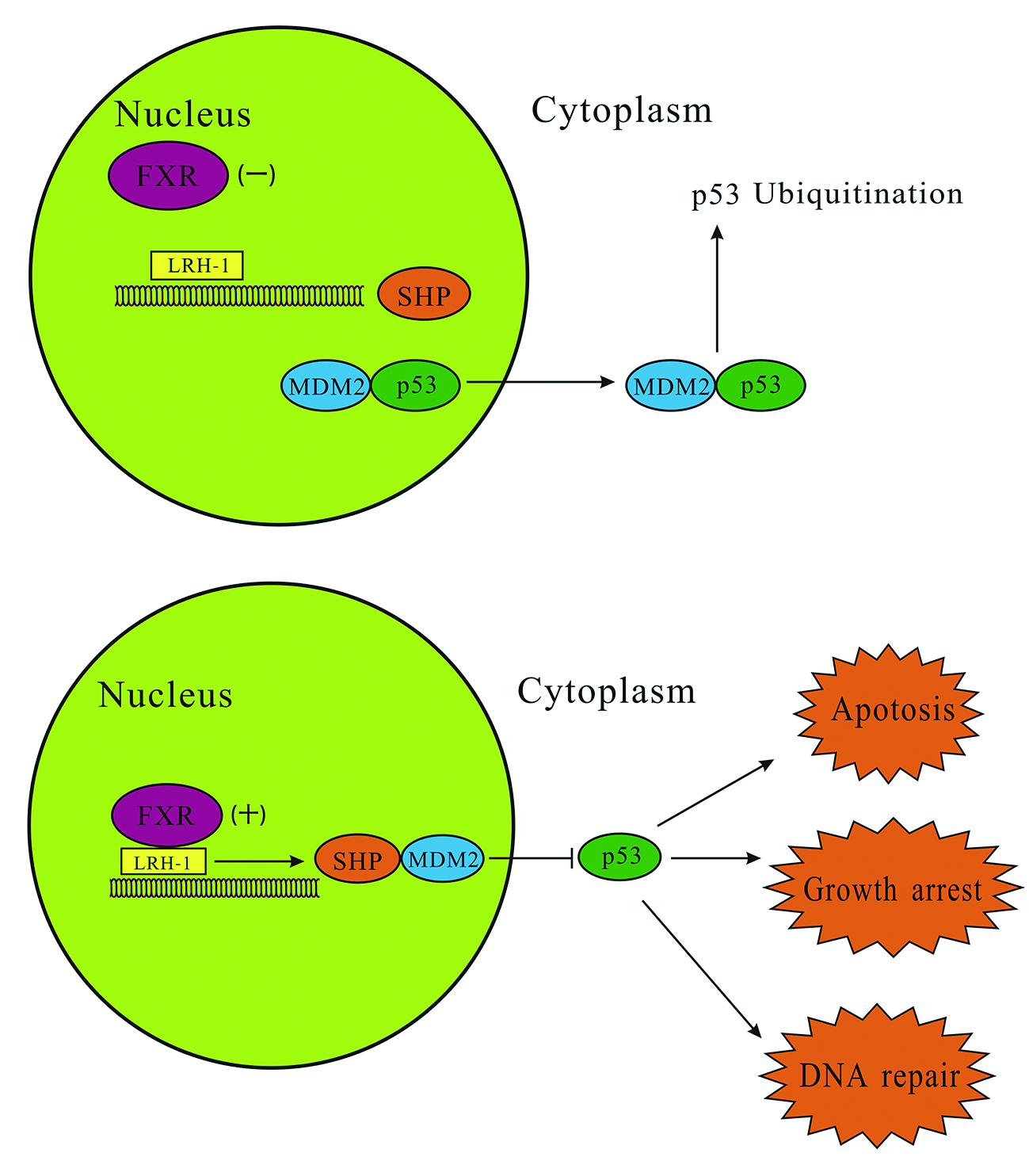

Supplement: Supplementary Figure 2 — The pathway of FXR-mediated p53 upregulation. [file Image_2.TIF]

## Slide 1
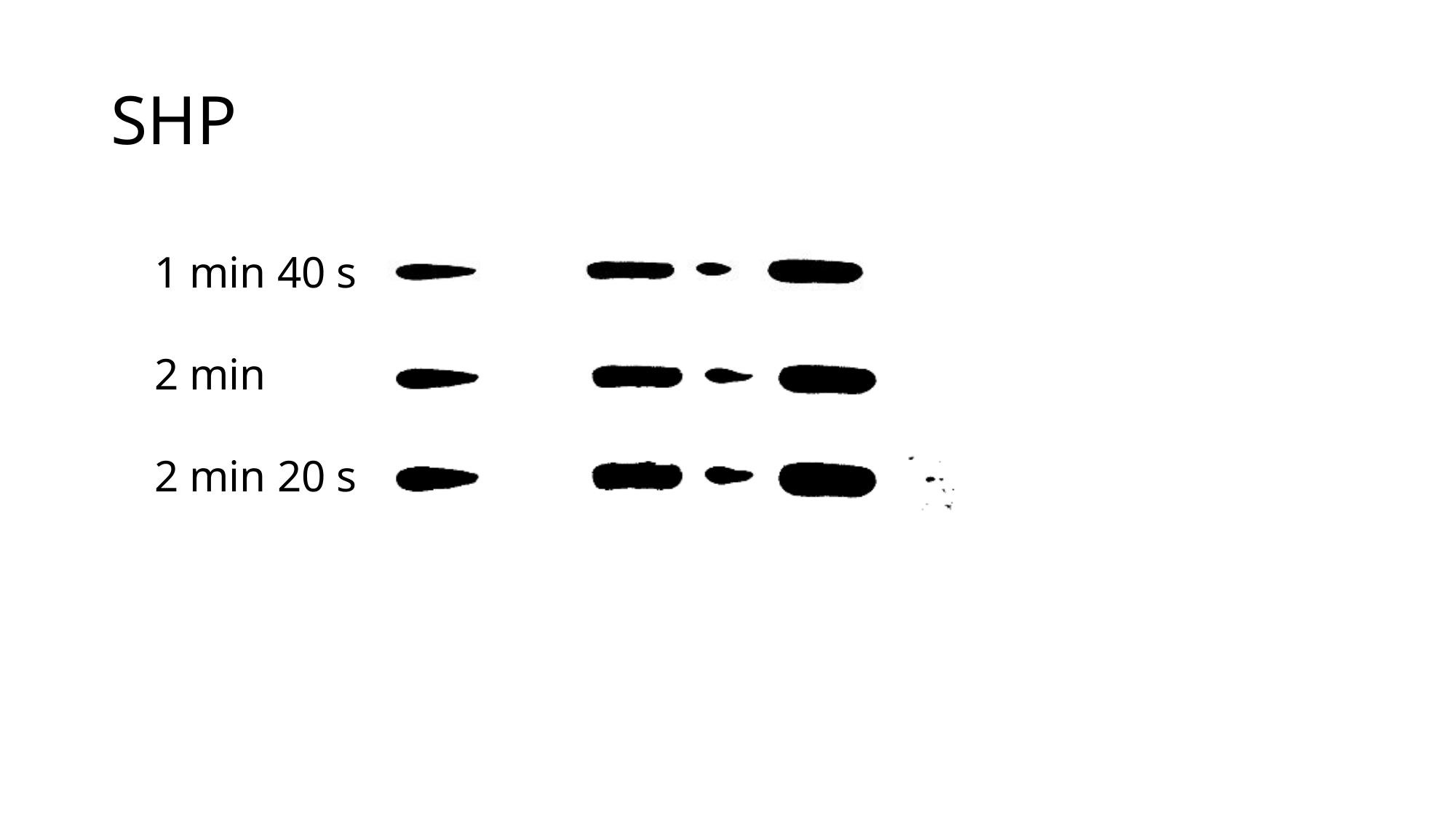

# SHP
1 min 40 s
2 min
2 min 20 s

## Slide 2
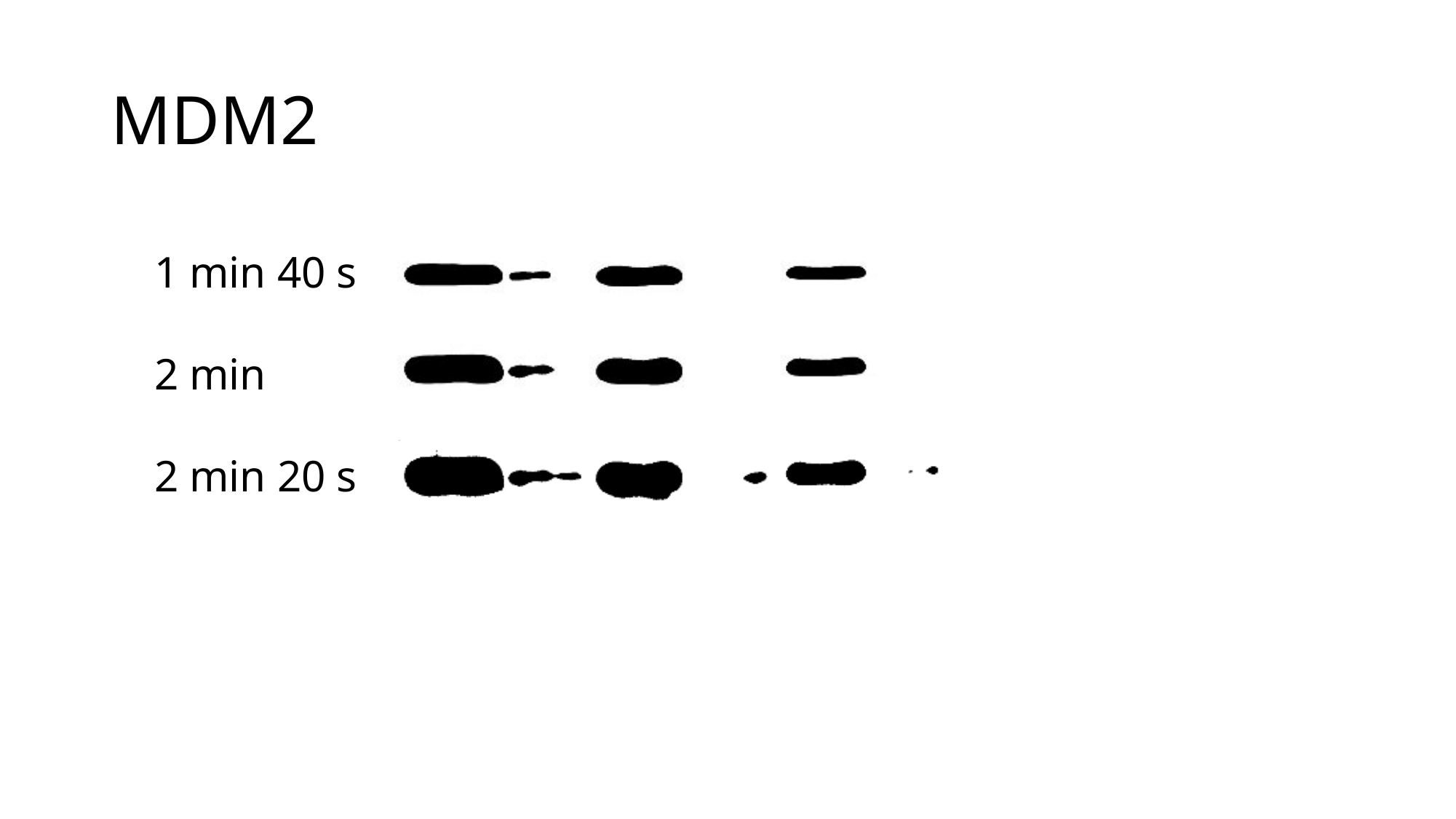

# MDM2
1 min 40 s
2 min
2 min 20 s

## Slide 3
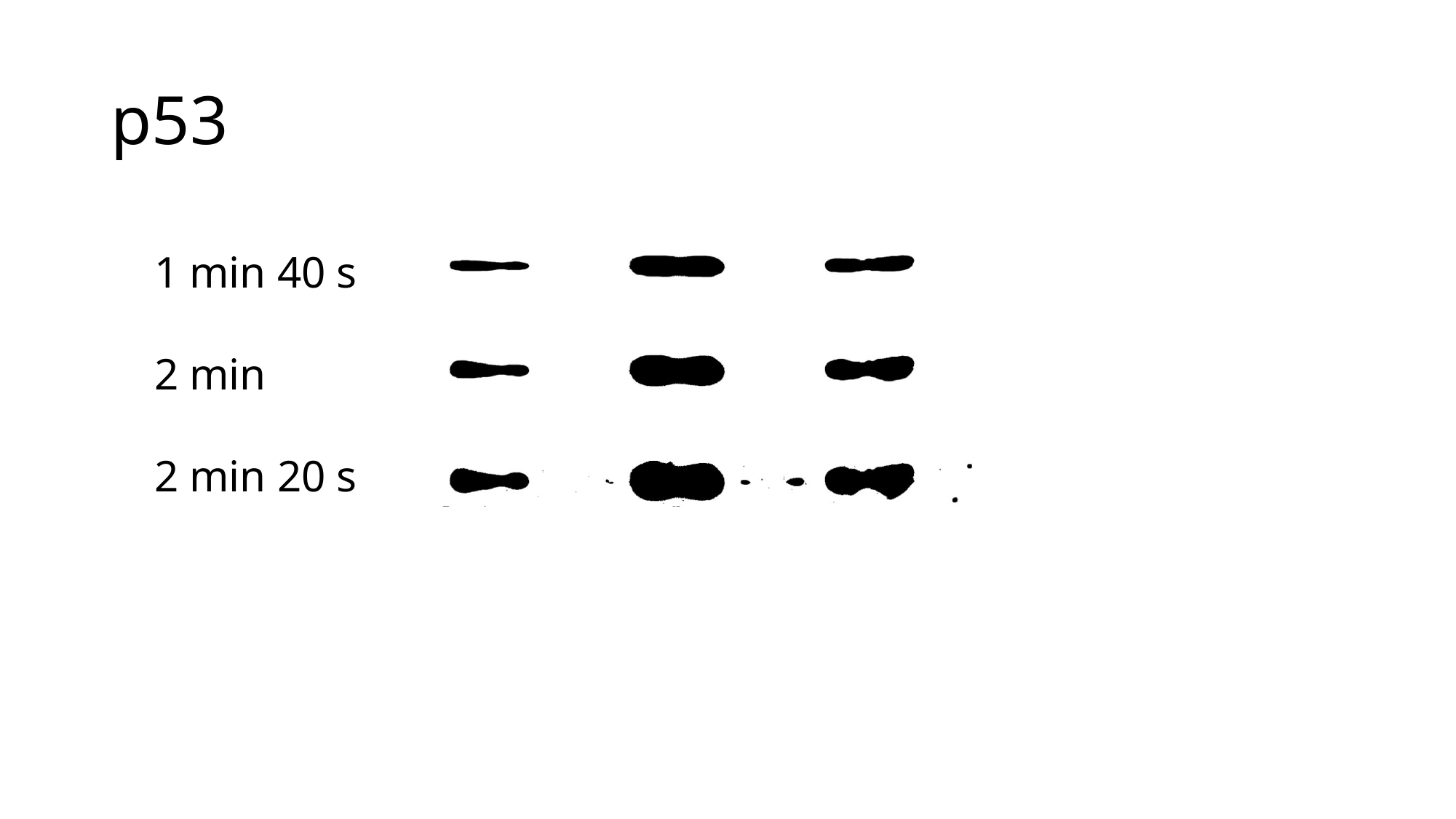

# p53
1 min 40 s
2 min
2 min 20 s

## Slide 4
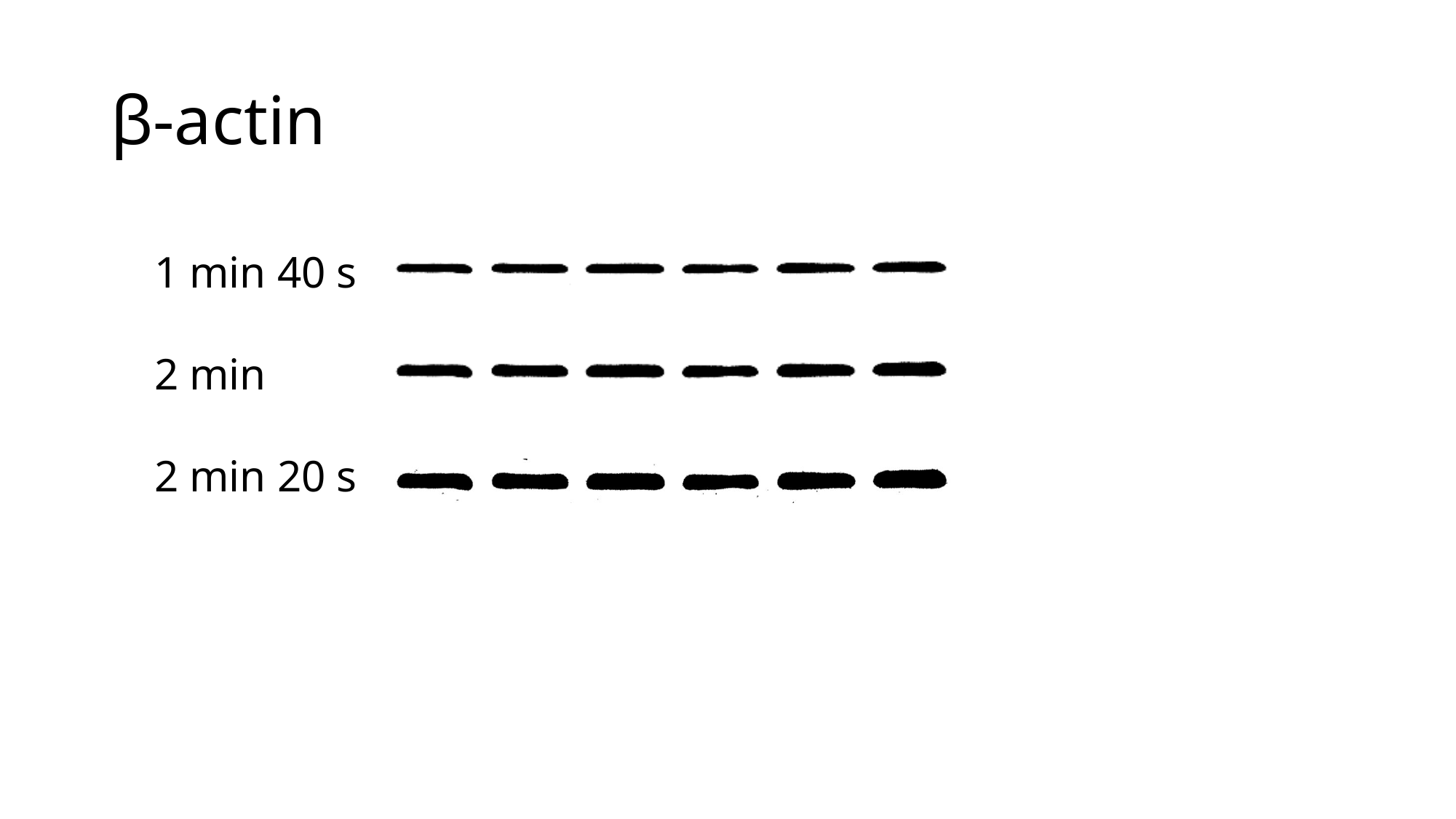

# β-actin
1 min 40 s
2 min
2 min 20 s

Supplement: Supplementary file 6 [file Presentation_2.PPTX]
