# Supplementary material for: The Nuclear Farnesoid X Receptor Reduces p53 Ubiquitination and Inhibits Cervical Cancer Cell Proliferation
Source: Front Cell Dev Biol. 2021 Apr 6;9:583146. doi: 10.3389/fcell.2021.583146 (PMC8056046; doi:10.3389/fcell.2021.583146)
Supplement: Supplementary file 3 [file Data_Sheet_1.doc]

Supplementary Table 1. Clinical features of cervical cancer patients

| Variable | Value |
| --- | --- |
| Total number  Median age (year)  Stage  SⅠb1  SⅠb2  SⅡa1  SⅡa2  SⅡb2  Lymph node metastasis  Negative  Positive  Tumor size (cm)  ≤4.0  >4.0 | 37  45.8  15  9  5  4  4  32  5  13  24 |
| Based on the FIGO 2009 criteria. |  |

Supplementary Table 2. Data of Tissue Microarray

| Variable | Value |
| --- | --- |
| Total number  Median age (year)  Normal cervical tissue  Cervical cancer  Stage  SⅠa  SⅠb  SⅡa  SⅡb  SⅢ | 165  44.2  13  152  9  65  35  30  13 |

Supplementary Table 3. Primer sequences

| Genes | Sense (5’-3’) | Antisense (5’-3’) |
| --- | --- | --- |
| β-actin | TTGCTGATCCACATCTGCT | GACAGGATGCAGAAGGA |
| FXR | GATTGCTTTGCTGAAAGGGTC | CAGAATGCCCAGACGGAAG |
| SHP | TCAAGTCCATTCCGACCAGC | AAGAAGGCCAGCGATGTCAA |

Supplementary Table 4. Data of antibodies

| Antibodies | Item No. | Company |
| --- | --- | --- |
| anti-β-actin | TA-09 | Zsbio |
| mouse anti-MDM2 | sc-965 | Santa Cruz |
| mouse anti-p53 | sc-98 | Santa Cruz |
| rabbit anti-FXR | sc-13063 | Santa Cruz |
| mouse anti-SHP | sc-271511 | Santa Cruz |
| HRP anti-rabbit IgG | BA1054 | Boster |
| HRP anti-mouse IgG | BA1050 | Boster |
| rabbit anti-MDM2 | SAB4501849 | Sigma |

Supplementary Table 5. Groups in Reporter assay

| No. | Names | Groups |
| --- | --- | --- |
| 1 | Lenti-FXR cell lines | pGL3-SHP + pRL-TK |
| 2 | Lenti-Vector cell lines | pGL3-SHP + pRL-TK |

Supplementary Table 6. Sequences of siRNA

| Genes | Sequences（5’-3’） | |
| --- | --- | --- |
| hs-SHP | sense  anti-sense | GCUGUGUGAAGUCCUGGAAdTdT  UUCCAGGACUUCACACAGCdTdT |

Supplementary Table 7. Data of antibodies in ubiquitination

| Antibodies | Item No. | Company |
| --- | --- | --- |
| rabbit anti-MDM2 | D160611 | Sangon Biotech |
| rabbit anti-p53 | D151468 | Sangon Biotech |
| rabbit anti-FXR | ab155124 | abcam |
| rabbit anti-Ubiquitin | ab7780 | Abcan |

Supplementary Table 8. Primer sequences of pcDNA3.1-SHP

| Primer | Forward (5’-3’) | Reverse (5’-3’) |
| --- | --- | --- |
| pcDNA3.1-SHP | CTCGAGAGGTTGAGAGGCTGGAGTGG | AGCTTCAAGCCAGGTGAGGAAGAACCGC |
