# Supplementary material for: The Nuclear Farnesoid X Receptor Reduces p53 Ubiquitination and Inhibits Cervical Cancer Cell Proliferation
Source: Front Cell Dev Biol. 2021 Apr 6;9:583146. doi: 10.3389/fcell.2021.583146 (PMC8056046; doi:10.3389/fcell.2021.583146)
Supplement: Supplementary file 4 [file Data_Sheet_2.docx]

**SUPPLEMENTARY MATERIALS AND METHODS**

# *RNA extraction and real-time quantitative PCR*

Tissue samples were ground in liquid nitrogen for RNA extraction using TRIzol (Takara, Japan). After washing with 75% alcohol, total RNA was dissolved in DEPC-treated water and quantified by spectrophotometry. The PrimeScript^TM^ reverse transcriptase kit (Takara, Japan) was used for reverse transcription. Real-time quantitative PCR was performed on an Applied Biosystems™ 7500 Real-Time PCR System (Applied Biosystems, USA) at 95°C for 5 s and 60°C for 34 s, for a total of 40 cycles. β-actin was used for normalization. The primer sequences are shown in Supplementary Table 3.

*Western blot*

After the addition of the RIPA lysis buffer (Beyotime, China), the ground tissue samples or cells were vortexed on ice. The lysates were centrifuged at 12,000 ×g for 5 min at 4°C, and the supernatants were transferred to clean tubes. Nuclear and cytoplasmic proteins were isolated with the Protein Extraction Kit (Sangon, China) on ice, according to the manufacturer’s instructions. The total protein was quantified with a Pierce^TM^ Protein BCA assay kit (Thermo, USA). Equal amounts of total protein were separated by 10%-12% SDS-PAGE and transferred onto nitrocellulose membranes (Boster, USA). The membranes were blocked at 37°C for 1 h, followed by incubation with mouse anti-β-actin, mouse anti-MDM2, mouse anti-p53, rabbit anti-FXR, and mouse anti-SHP primary antibodies, at 4°C overnight. HRP anti-rabbit and HRP anti-mouse IgG were used as secondary antibodies, as appropriate (Supplementary Table 4).

*Immunohistochemistry*

Tissue sections were dewaxed with xylene and dehydrated with different concentrations of ethanol. After incubation with H_2_O_2_, antigen retrieval with antigen repair solution (Beyotime, China), and blocking with 5% BSA (Boster, USA), the tissue sections were incubated with mouse anti-MDM2, mouse anti-p53 and rabbit anti-FXR primary antibodies (Supplementary Table 4) at 4°C overnight. After washing with PBS, incubation with secondary antibodies was performed. All slides were examined under an Olympus microscope. The Image-Pro Plus 6 software was used for quantification.

*Cell proliferation*

Cells were inoculated at a density of 3×10^3^ cells/well in 96-well plates. CA (100 µg/mL) ([1](#_ENREF_1)), LCA (40 µmol/L) ([2](#_ENREF_2)), CDCA (50 µmol/L) ([3](#_ENREF_3), [4](#_ENREF_4)), and GW4064 (2 µmol/L) ([3](#_ENREF_3)) were added to the medium, respectively, and cell viability was measured by the MTT (Sigma, USA) assay at 24, 48, and 72 h. In addition, the Lenti-FXR and Lenti-Vector groups of CaSki, SiHa, and HeLa cells were assessed for viability as described above. After treatment, MTT was added to the medium, and the plates were incubated at 37°C for 4 h. Then, DMSO (Sigma, USA) was added to each well, and absorbance was read at 490 nm on a microplate reader (SpectraMax, USA).

*Colony formation assay*

Cells were inoculated at 500 cells/ml/well, administered CDCA (50 µmol/l) after overnight culture, and incubated for an additional 2 weeks. After washing with PBS, the cells were fixed with 4% paraformaldehyde and stained with Giemsa. Clones were counted with the Image-Pro Plus 6 software. The colony formation rate was assessed as (colony number/cell inoculation number) ×100%.

*Flow cytometry*

Cells (1×10^6^) were harvested and washed with cold PBS, followed by fixation with 70% ethanol overnight. After washing with PBS, the cells were incubated with 0.5 ml PBS containing 100 U/ml propidium iodide (PI, Sigma, USA) and RNase A (50 μg/ml). The cells were analyzed on a BD Accuri^TM^ C6 with the 7.6 Flowjo software.

*Apoptosis assessment*

Cells were collected, washed with cold PBS, centrifuged, and stained with Annexin-V-FITC (0.25 µg/ml, Dojindo, Japan) and PI for 15 min at room temperature in the dark. The stained cells were analyzed by flow cytometry, acquiring 1×10^5^ events gated according to a large gate established on cell forward and side scatters within 30 min of staining.

*Reporter assay*

Lenti-FXR and Lenti-Vector cells were transiently transfected with the SHP promoter-luciferase reporter plasmid (Supplementary Fig. 1A), containing the LRH-1 binding site, and pRL-TK in 24-well plates (Supplementary Table 5). The activities of the Firefly and Renilla luciferase reporters were measured at 48 h after transfection with the Dual Luciferase Assay kit (Promega, USA). TOP-Flash reporter activity was derived as the relative ratio of firefly luciferase activity to Renilla luciferase activity.

*Vector transfection*

si-SHP (Biotend, China) was transfected into CaSki, SiHa, and HeLa cells using the Lipofectamine 2000 reagent (Invitrogen, USA). RNA was extracted 24 h after transfection, and total protein was extracted 48 h after transfection. Sequences are shown in Supplementary Table 6.

*Co-immunoprecipitation*

HEK293T cells were transfected using Lipofectamine 2000 (Invitrogen, USA) and lysed with lysis buffer. Anti-FLAG M2 magnetic beads (Sigma, M8823, USA; 50 µl/sample) were used for immunoprecipitation. The beads were washed four times with lysis buffer. The conjugated proteins were separated by SDS-PAGE followed by western blot with primary antibodies, including rabbit anti-MDM2, rabbit anti-p53, rabbit anti-FXR, and rabbit anti-ubiquitin (Supplementary Table 7).

In another assay, Lenti-FXR CaSki cells were washed with PBS and lysed with lysis buffer on ice. The amounts of total protein were measured with a Pierce^TM^ Protein BCA assay kit (Thermo, USA). The supernatants were incubated with 2 μg IgG or mouse anti-SHP (Santa Cruz, USA). The mixture was incubated with protein G-Agarose beads (Roche, USA) at 4°C overnight. The beads were washed four times with lysis buffer. Then, 2× SDS buffer was added and boiled for 5 min, and samples were assessed by western blot.

*Immunofluorescence*

Cells were transfected with si-SHP or treated with Nutlin-3a. At 24 h, they were fixed with 4% paraformaldehyde, permeabilized with 0.3% Triton X-100 and incubated with mouse anti-MDM2, mouse anti-p53, and mouse anti-SHP primary antibodies, respectively (Supplementary Table 4). After washing with PBS, the cells were incubated with fluorescent secondary antibodies for 1 h. The cells were counterstained with 4',6-diamidino-2-phenylindole (DAPI) and observed under a Leica inverted fluorescence microscope.

**REFERENCES**

1. H. K. Chong, A. M. Infante, Y. K. Seo, T. I. Jeon, Y. Zhang, P. A. Edwards, X. Xie and T. F. Osborne: Genome-wide interrogation of hepatic FXR reveals an asymmetric IR-1 motif and synergy with LRH-1. *Nucleic Acids Res*, 38(18), 6007-17 (2010) doi:10.1093/nar/gkq397

2. M. O. Hoeke, J. Heegsma, M. Hoekstra, H. Moshage and K. N. Faber: Human FXR regulates SHP expression through direct binding to an LRH-1 binding site, independent of an IR-1 and LRH-1. *PLoS One*, 9(2), e88011 (2014) doi:10.1371/journal.pone.0088011

3. J. C. Marine and G. Lozano: Mdm2-mediated ubiquitylation: p53 and beyond. *Cell Death Differ*, 17(1), 93-102 (2010) doi:10.1038/cdd.2009.68

4. J. J. Manfredi: The Mdm2-p53 relationship evolves: Mdm2 swings both ways as an oncogene and a tumor suppressor. *Genes Dev*, 24(15), 1580-9 (2010) doi:10.1101/gad.1941710
