# Supplementary material for: The Nuclear Farnesoid X Receptor Reduces p53 Ubiquitination and Inhibits Cervical Cancer Cell Proliferation
Source: Front Cell Dev Biol. 2021 Apr 6;9:583146. doi: 10.3389/fcell.2021.583146 (PMC8056046; doi:10.3389/fcell.2021.583146)
Supplement: Supplementary file 5 [file Presentation_1.PPTX]

## Slide 1
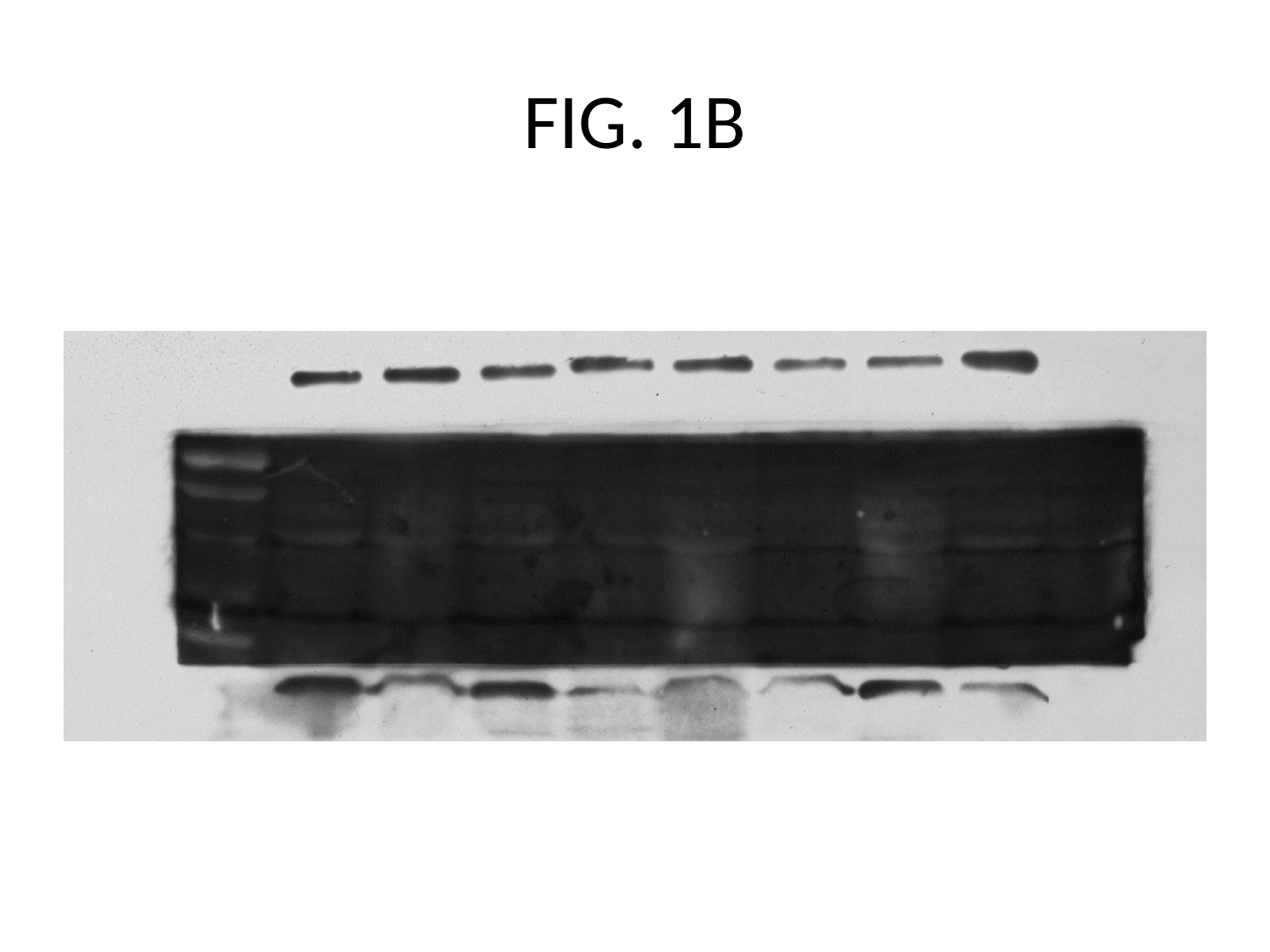

# FIG. 1B

## Slide 2
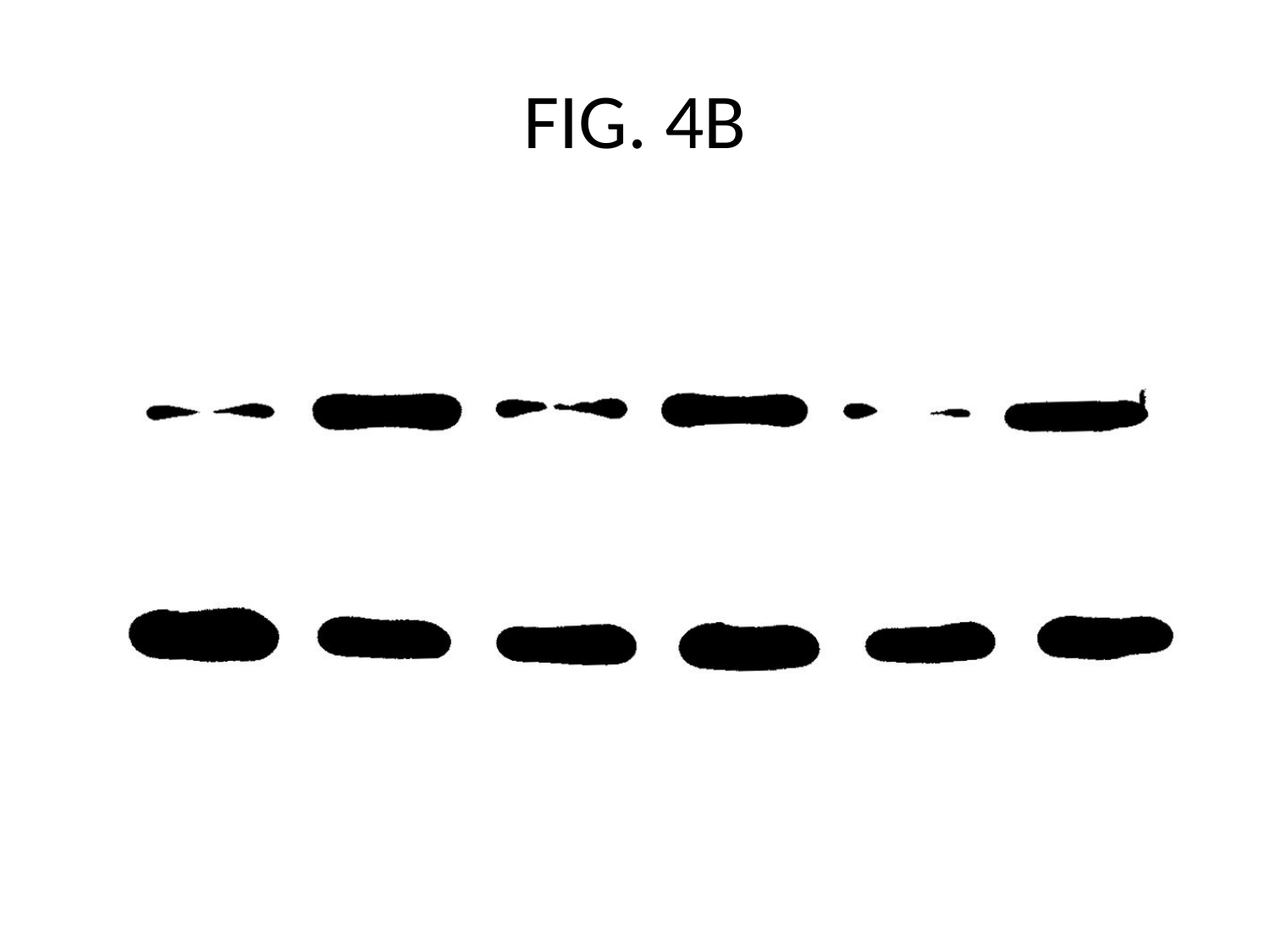

# FIG. 4B

## Slide 3
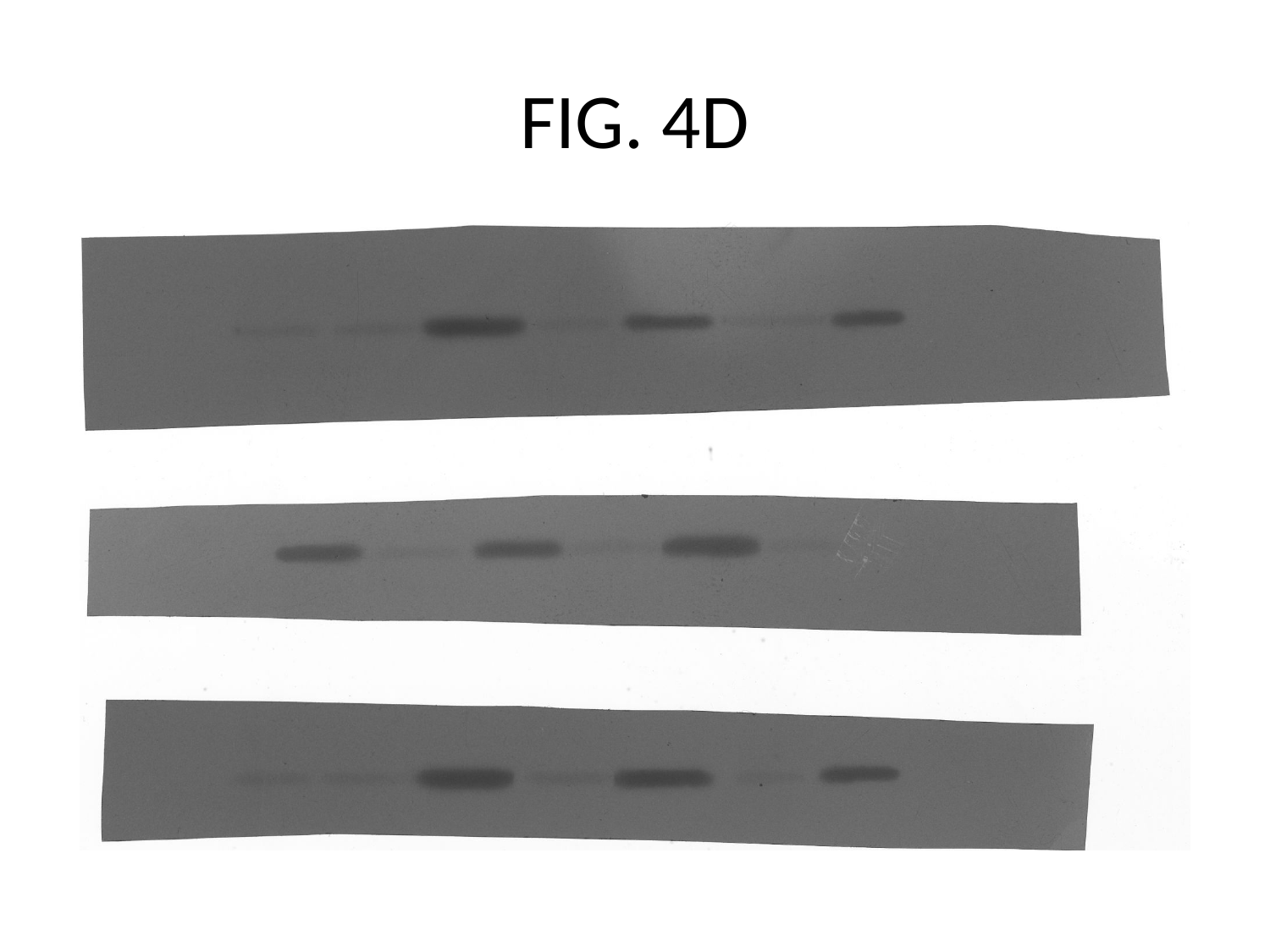

# FIG. 4D

## Slide 4
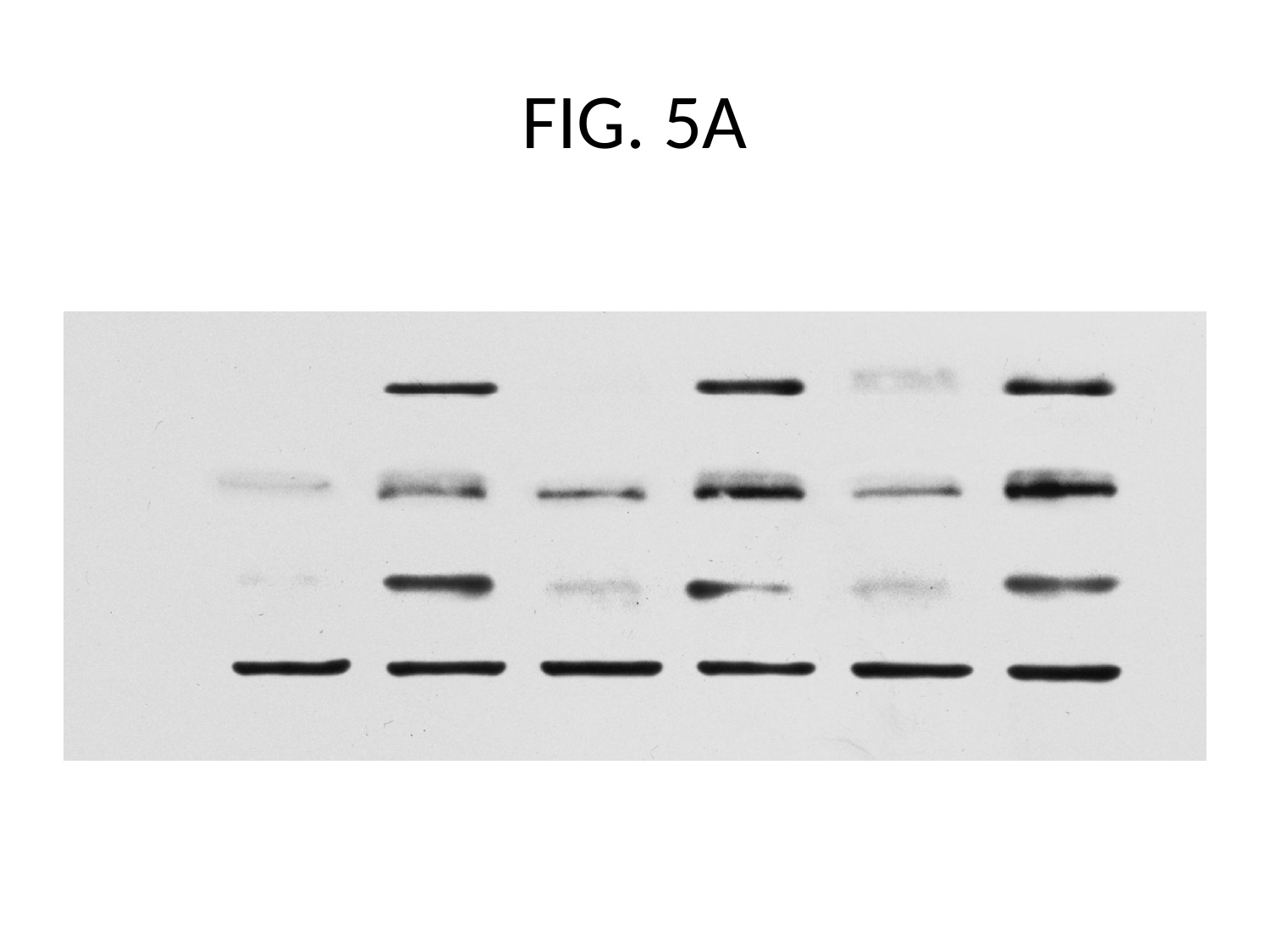

# FIG. 5A

## Slide 5
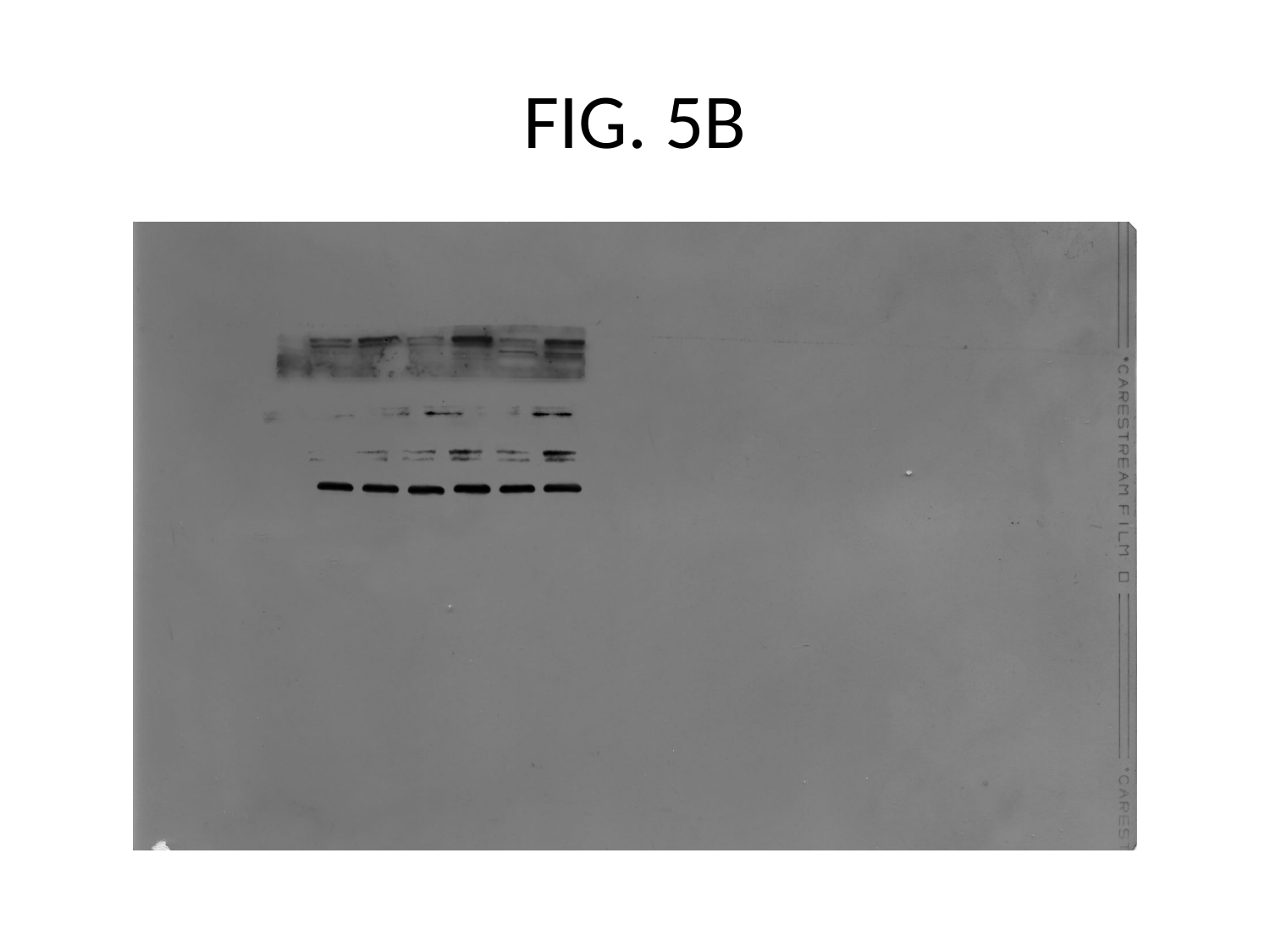

# FIG. 5B

## Slide 6
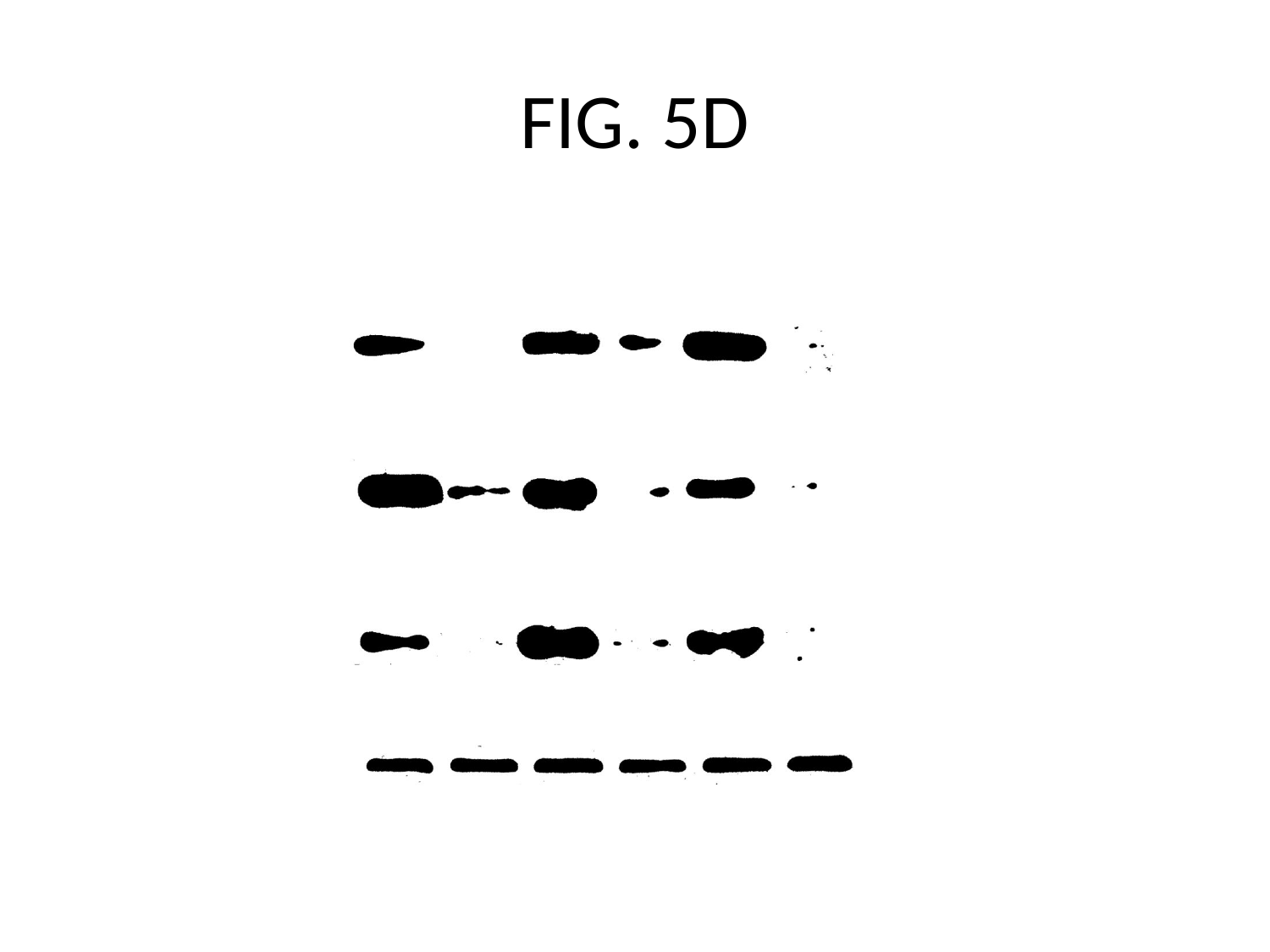

# FIG. 5D

## Slide 7
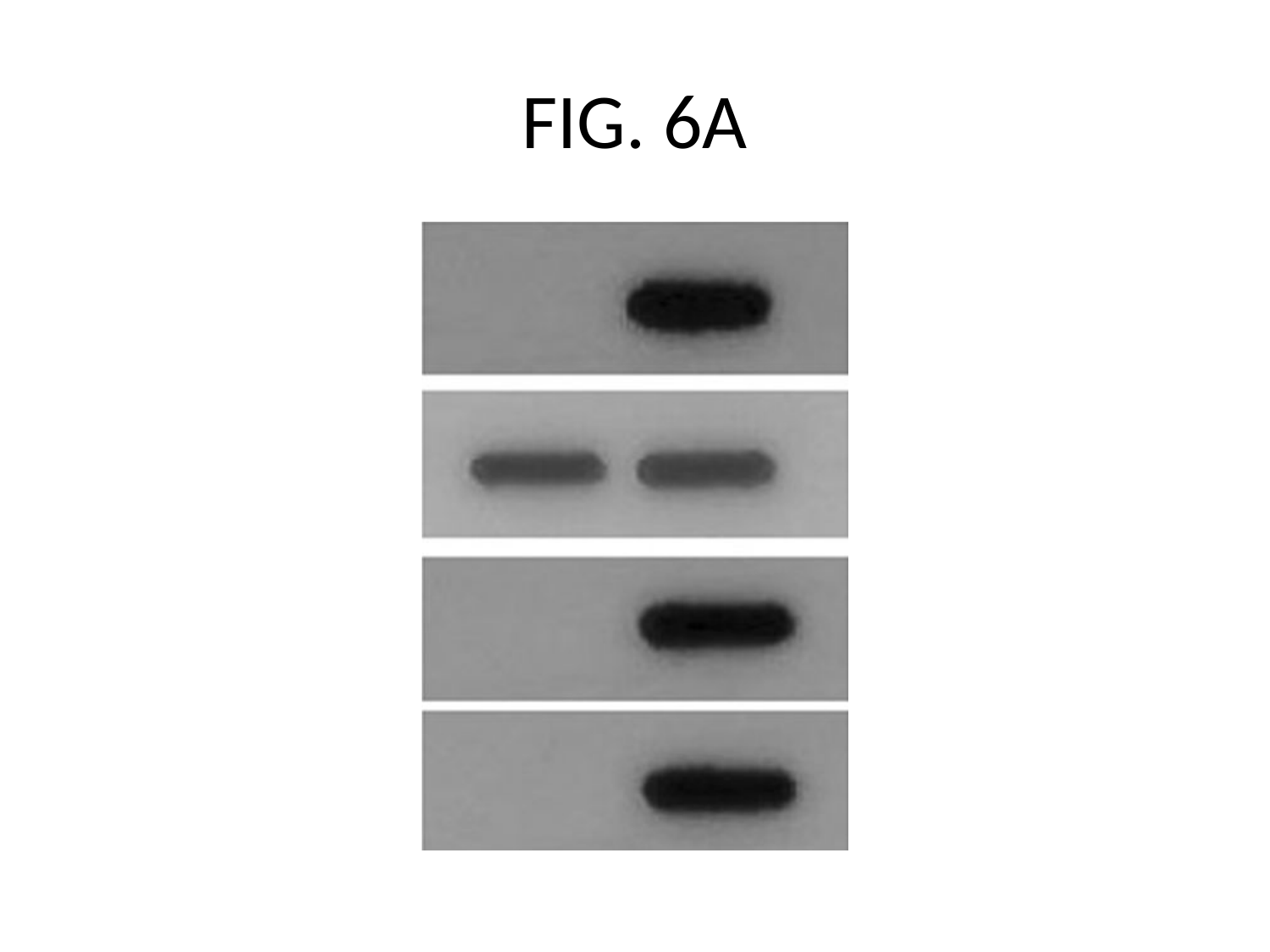

# FIG. 6A

## Slide 8
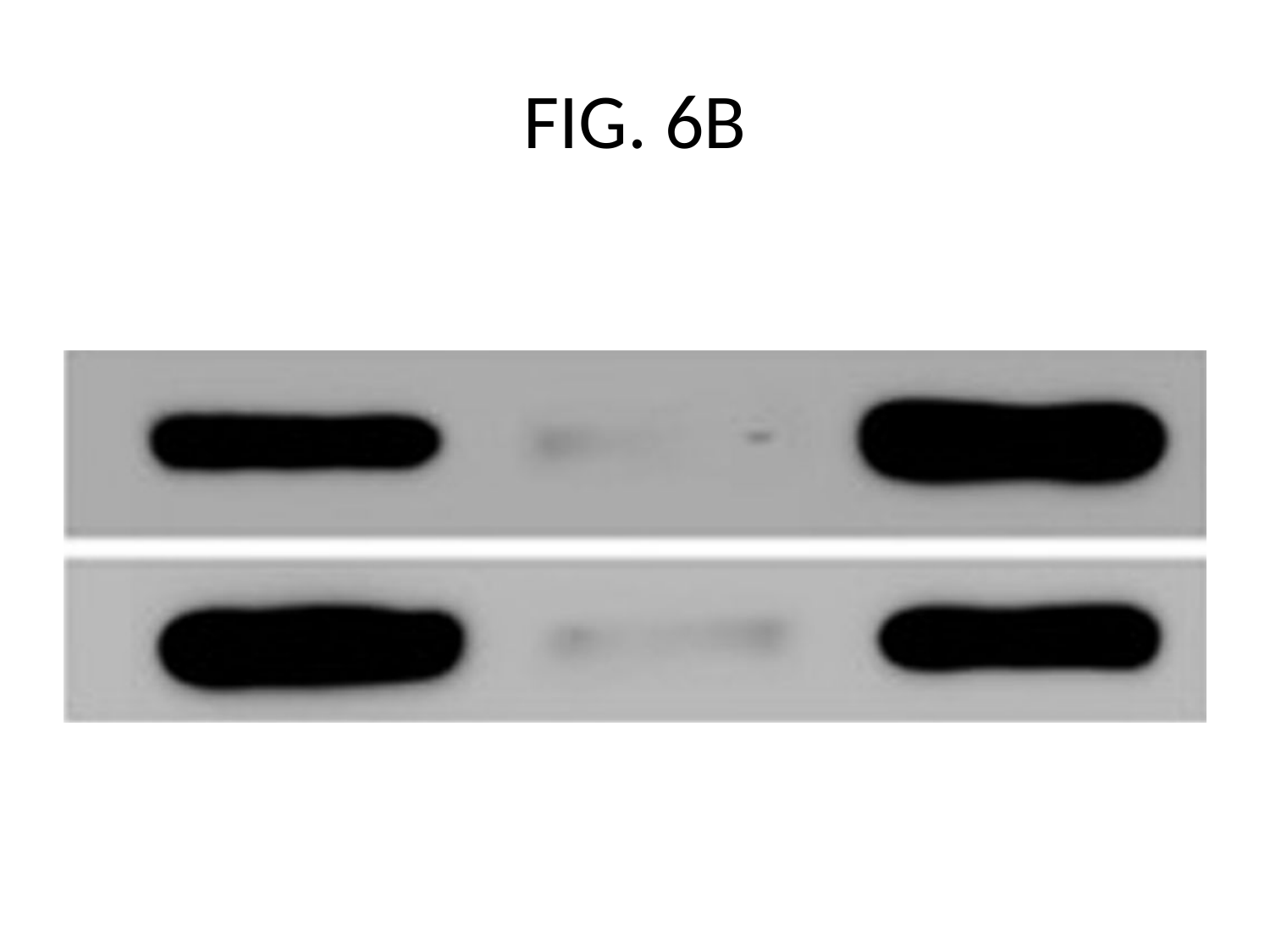

# FIG. 6B

## Slide 9
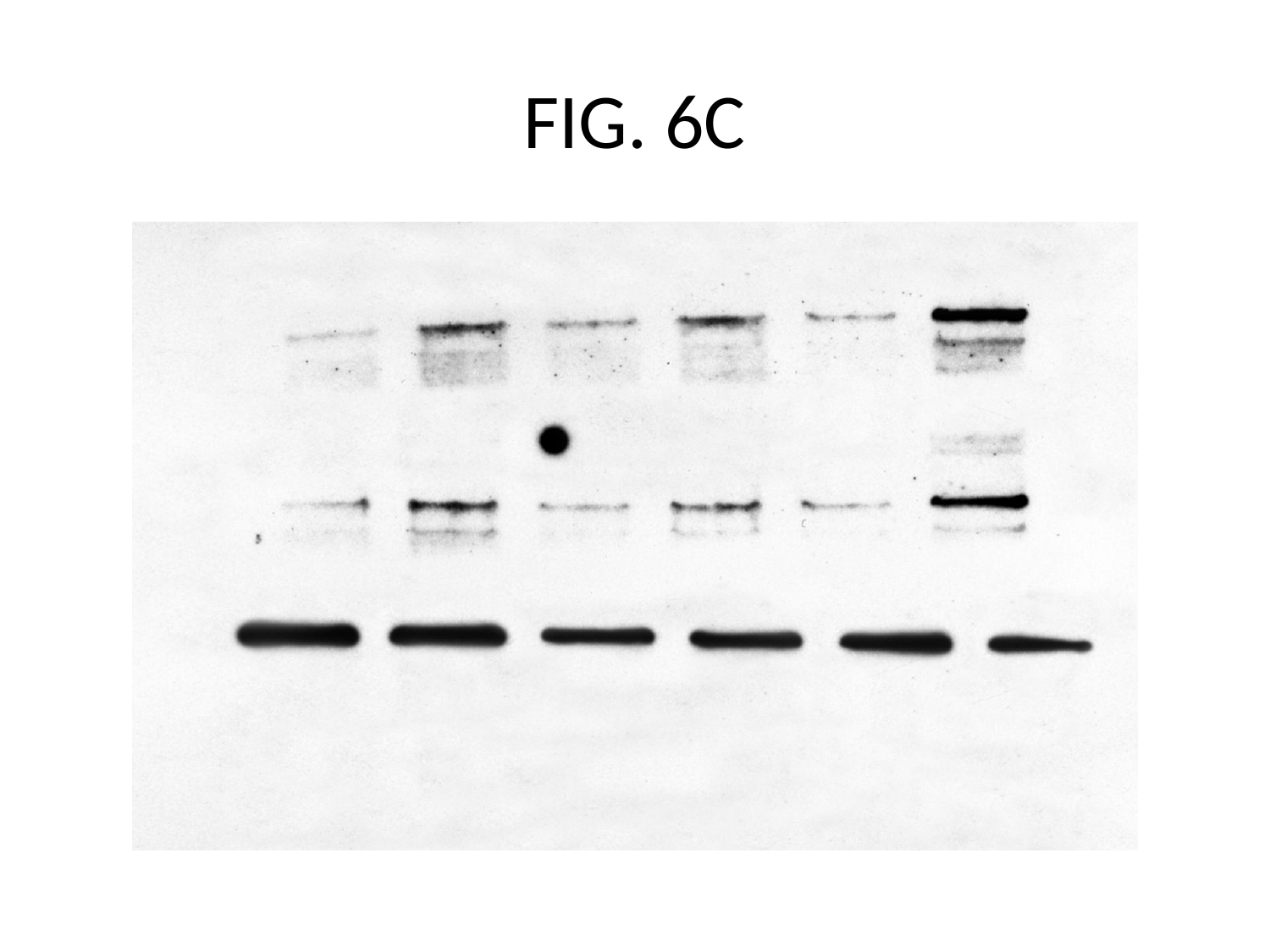

# FIG. 6C

## Slide 10
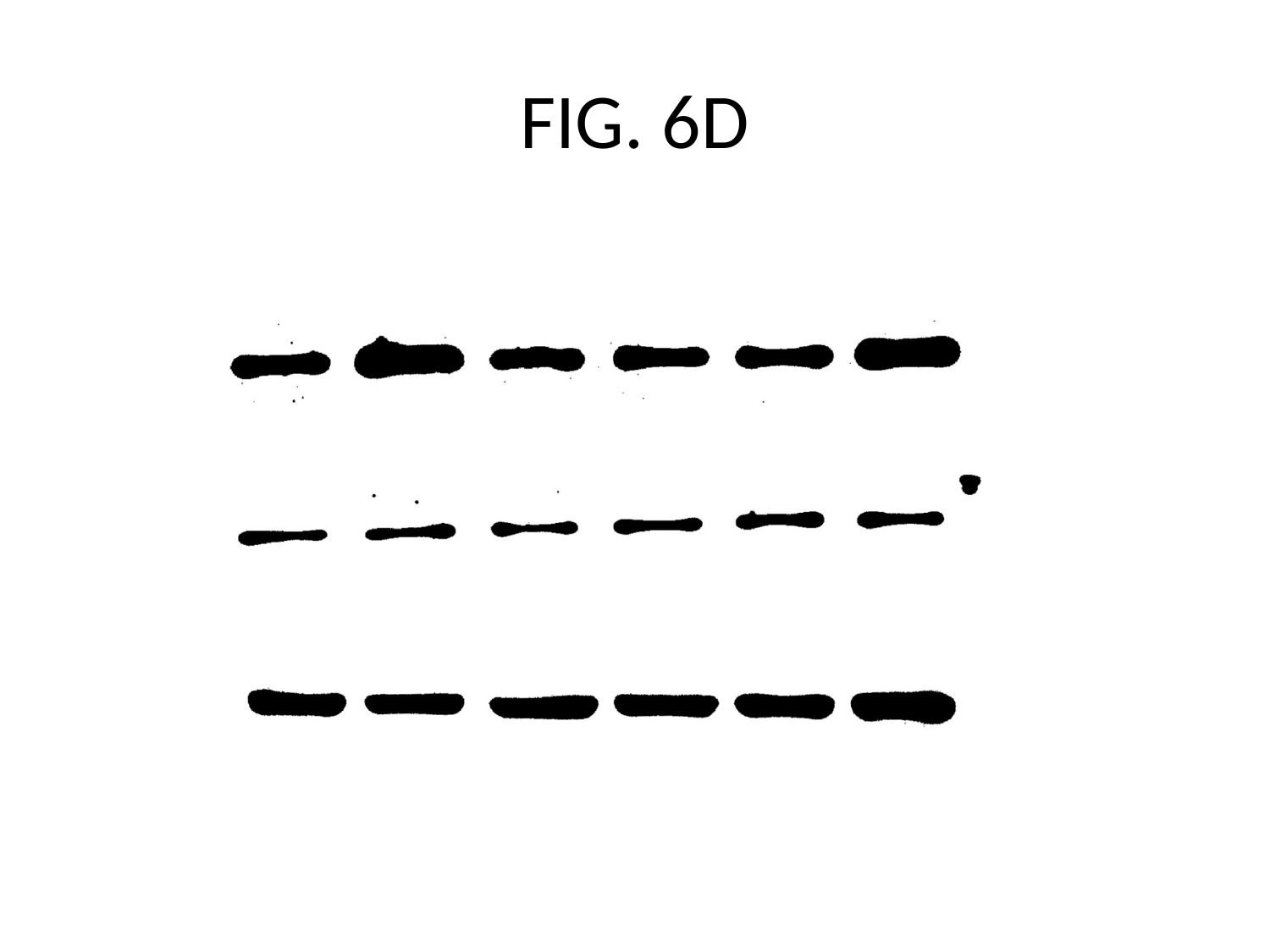

# FIG. 6D

## Slide 11
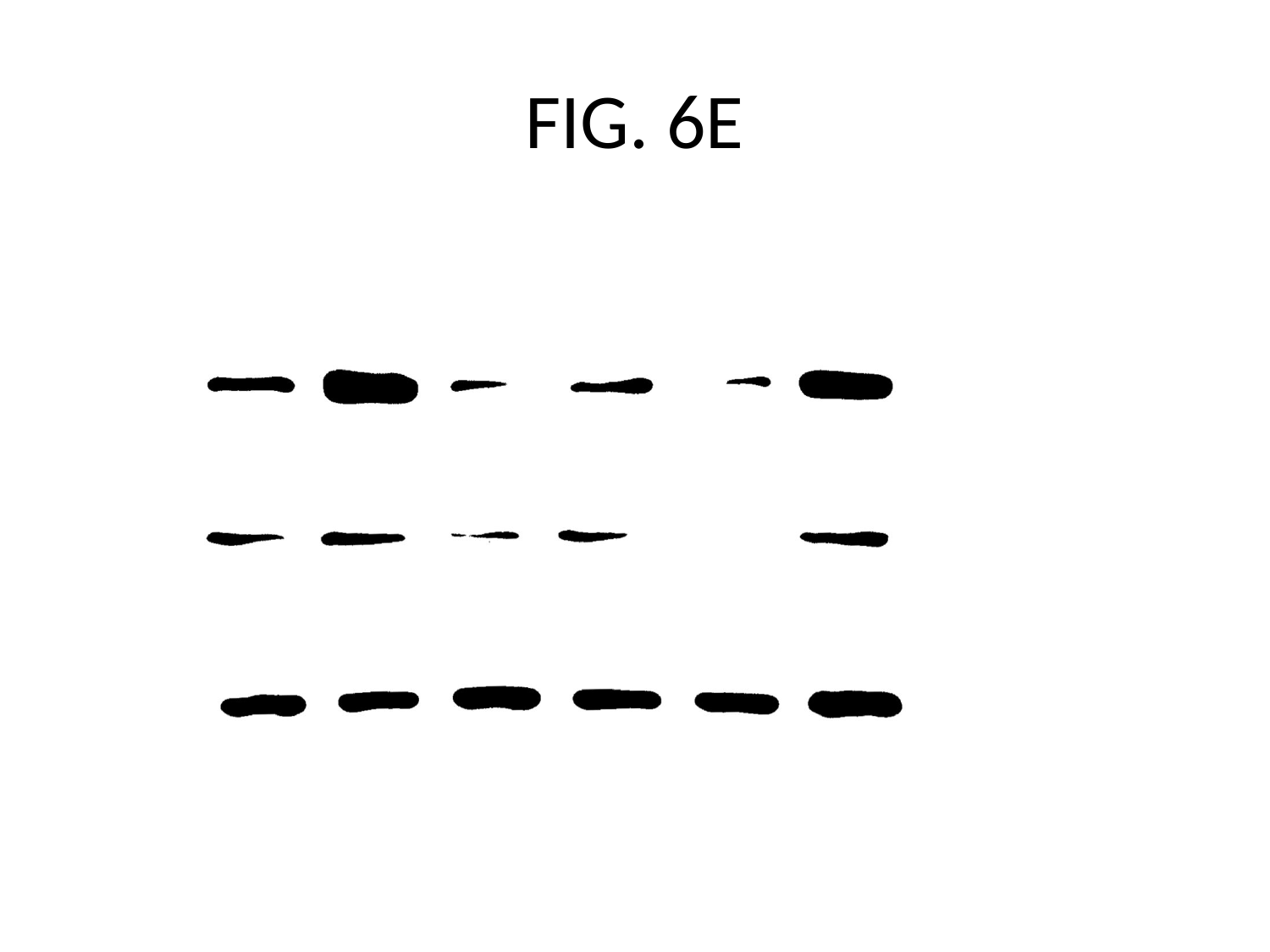

# FIG. 6E

## Slide 12
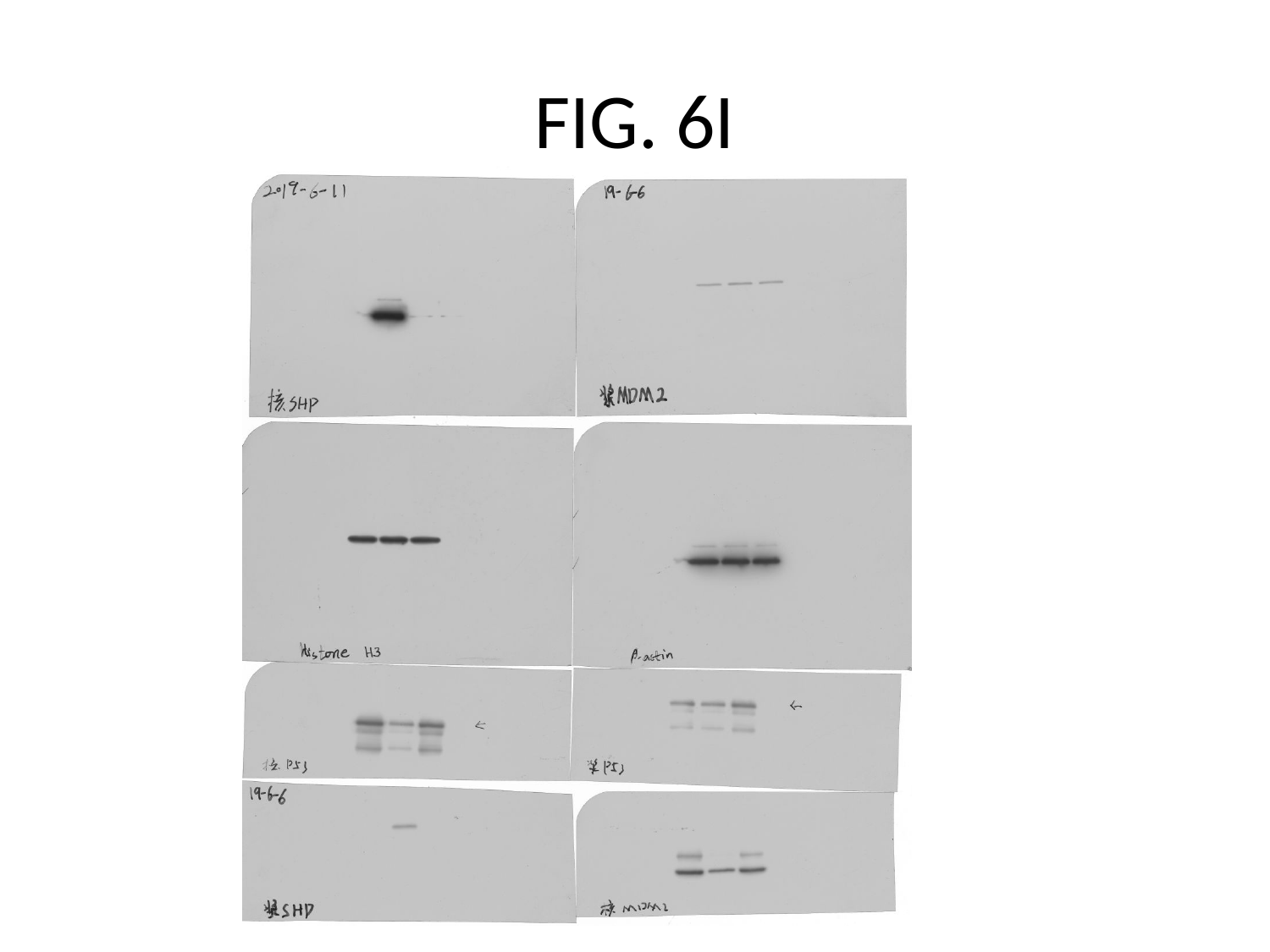

# FIG. 6I

## Slide 13
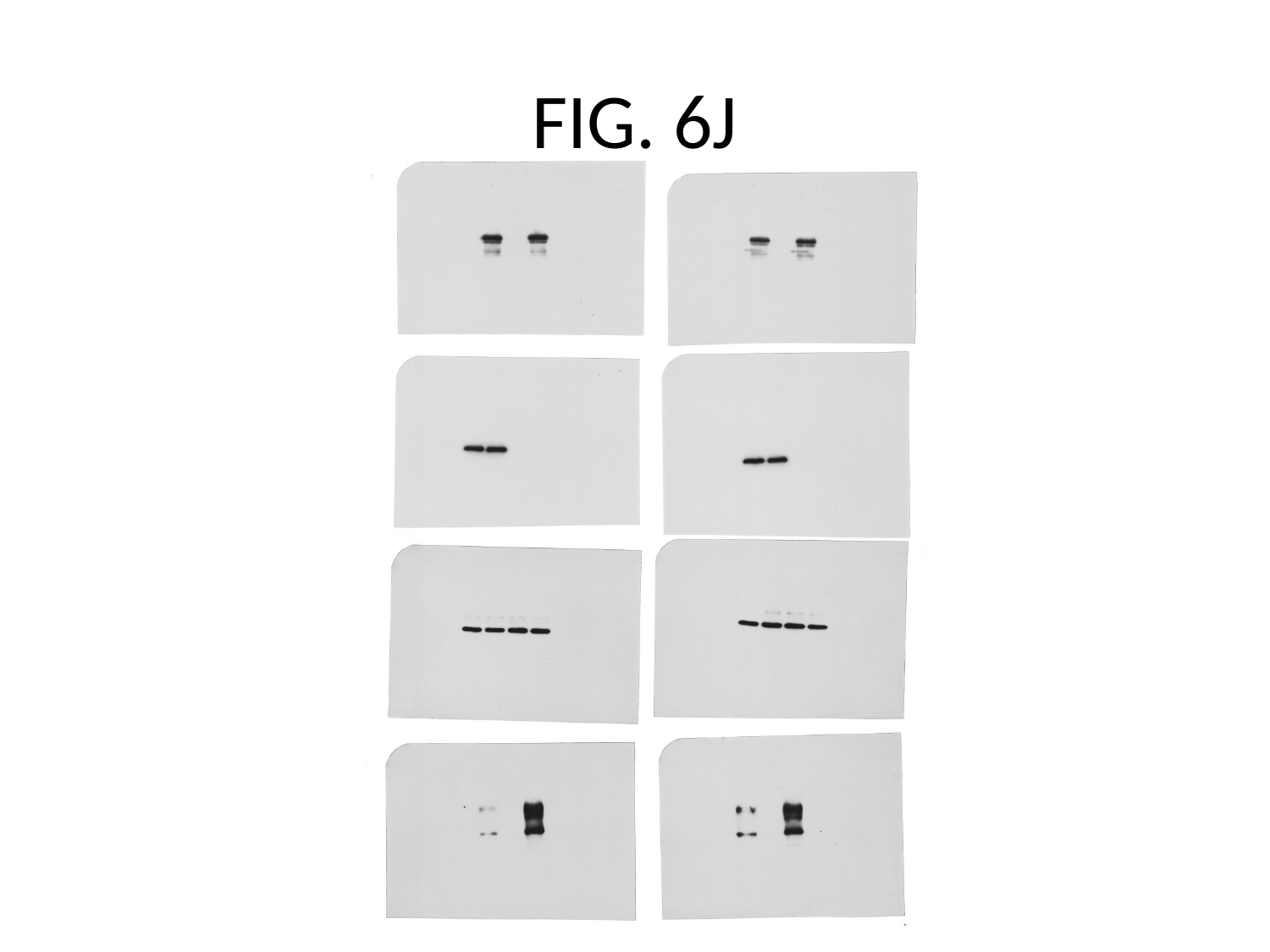

# FIG. 6J
